# Therapeutic Targets for Heart Failure Identified Using Proteomics and Mendelian Randomization

**DOI:** 10.1161/CIRCULATIONAHA.121.056663

**Published:** 2022-03-18

**Authors:** Albert Henry, María Gordillo-Marañón, Chris Finan, Amand F. Schmidt, João Pedro Ferreira, Ravi Karra, Johan Sundström, Lars Lind, Johan Ärnlöv, Faiez Zannad, Anders Mälarstig, Aroon D. Hingorani, R. Thomas Lumbers, HERMES and SCALLOP Consortia

**Affiliations:** 1 Institute of Cardiovascular Science (A.H., M.G.-M., C.F., A.F.S., A.D.H.), University College London, United Kingdom.; 2 British Heart Foundation Research Accelerator (A.H., M.G.-M., C.F., A.F.S., A.D.H., R.T.L.), University College London, United Kingdom.; 3 Institute of Health Informatics (A.H., R.T.L.), University College London, United Kingdom.; 4 Health Data Research UK London (R.T.L.), University College London, United Kingdom.; 5 Department of Cardiology, Division of Heart and Lungs, University Medical Center Utrecht, The Netherlands (C.F., A.F.S.).; 6 Unidade de Investigação e Desenvolvimento Cardiovascular, Rede de Investigação em Saúde, Department of Surgery and Physiology, Faculty of Medicine of the University of Porto, Portugal (J.P.F.).; 7 Université de Lorraine, Inserm, Centre d’Investigations Cliniques - Plurithématique 14-33, and Inserm U1116, Centre Hospitalier Régional Universitaire, French Clinical Research Infrastructure Network, Investigation Network Initiative - Cardiovascular and Renal Clinical Trialists, Nancy, France (J.P.F., F.Z.).; 8 Division of Cardiology, Department of Medicine (R.K.), Duke University Medical Center, Durham, NC.; 9 Department of Pathology (R.K.), Duke University Medical Center, Durham, NC.; 10 Department of Medical Sciences, Uppsala University, Sweden (J.S., L.L.).; 11 The George Institute for Global Health, University of New South Wales, Sydney, Australia (J.S.).; 12 School of Health and Social Studies, Dalarna University, Falun, Sweden (J.Ä.).; 13 Division of Family Medicine and Primary Care, Department of Neurobiology, Care Science and Society, Karolinska Institutet, Huddinge, Sweden (J.Ä.).; 14 Department of Medical Epidemiology and Biostatistics, Karolinska Institute, Solna‚ Sweden (A.M.).; 15 Emerging Science and Innovation, Pfizer Worldwide Research, Development and Medical, Cambridge, MA (A.M.).

**Keywords:** drug target prediction, heart failure, Mendelian randomization analysis, proteomics

## Abstract

Heart failure (HF) is a highly prevalent disorder for which disease mechanisms are incompletely understood. The discovery of disease-associated proteins with causal genetic evidence provides an opportunity to identify new therapeutic targets.

**Methods:**

We investigated the observational and causal associations of 90 cardiovascular proteins, which were measured using affinity-based proteomic assays. First, we estimated the associations of 90 cardiovascular proteins with incident heart failure by means of a fixed-effect meta-analysis of 4 population-based studies, composed of a total of 3019 participants with 732 HF events. The causal effects of HF-associated proteins were then investigated by Mendelian randomization, using *cis*-protein quantitative loci genetic instruments identified from genomewide association studies in more than 30 000 individuals. To improve the precision of causal estimates, we implemented an Mendelian randomization model that accounted for linkage disequilibrium between instruments and tested the robustness of causal estimates through a multiverse sensitivity analysis that included up to 120 combinations of instrument selection parameters and Mendelian randomization models per protein. The druggability of candidate proteins was surveyed, and mechanism of action and potential on-target side effects were explored with cross-trait Mendelian randomization analysis.

**Results:**

Forty-four of ninety proteins were positively associated with risk of incident HF (*P*<6.0×10^–4^). Among these, 8 proteins had evidence of a causal association with HF that was robust to multiverse sensitivity analysis: higher CSF-1 (macrophage colony-stimulating factor 1), Gal-3 (galectin-3) and KIM-1 (kidney injury molecule 1) were positively associated with risk of HF, whereas higher ADM (adrenomedullin), CHI3L1 (chitinase-3-like protein 1), CTSL1 (cathepsin L1), FGF-23 (fibroblast growth factor 23), and MMP-12 (matrix metalloproteinase-12) were protective. Therapeutics targeting ADM and Gal-3 are currently under evaluation in clinical trials, and all the remaining proteins were considered druggable, except KIM-1.

**Conclusions:**

We identified 44 circulating proteins that were associated with incident HF, of which 8 showed evidence of a causal relationship and 7 were druggable, including adrenomedullin, which represents a particularly promising drug target. Our approach demonstrates a tractable roadmap for the triangulation of population genomic and proteomic data for the prioritization of therapeutic targets for complex human diseases.

**Clinical Perspective**

**What Is New?**

- Among 90 proteins investigated for their association with heart failure onset, 44 were observationally associated, and 8 were causally associated, 2 of which are the target of drugs in early clinical trials for heart failure.
- Targeting adrenomedullin was estimated to protect against new-onset heart failure consistent with the agonist effect of adrenomedullin drug antibodies, which are under evaluation in clinical trials.

**What Are the Clinical Implications?**

- Findings provide confirmatory evidence for the development and evaluation of therapeutics targeting galectin-3 and adrenomedullin, which are currently being pursued in clinical trials for heart failure.
- Integrating population-scale genomic and proteomic data through triangulation of observational and Mendelian randomization analyses facilitates prioritization of drug targets and provides insights into molecular mechanisms of a complex clinical syndrome.

Heart failure (HF) is a clinical syndrome arising from disease processes that either injure or overload the heart muscle leading to inadequate function at normal filling pressures.^1^ Despite primary prevention through treatment of known antecedent risk factors, the prevalence is rising, and the burden of associated morbidity and mortality remains high.^2^ The challenge of recapitulating a complex age-associated disease entity such as HF in model systems is reflected in a history of late-stage failures of new therapeutics in clinical trials.^3–5^ More robust approaches to drug target identification and validation for HF are therefore required.^5^

Proteins are frequently the principal regulators of molecular pathways and the target of the majority of drugs.^6^ The circulating proteome is composed of proteins derived from almost all cells and tissues, which are either actively or passively secreted into the circulation or released during cell damage or turnover.^7^ Studies of the human circulating proteome measured using affinity or aptamer-based multiplexed assays have identified a large number of circulating proteins associated with HF onset, progression, and recovery.^8–10^ However, the causal relevance of associations from these nonrandomized, observational studies (referred to as observational associations in the present article) remains largely undetermined; they may arise because of confounding factors, reverse causation, or inclusion of undetected or asymptomatic prevalent cases at the time of protein measurement.

Mendelian randomization (MR) can be used to estimate the causal effect of protein levels on disease outcomes,^11^ on the condition that 3 core assumptions are met: that genetic instrumental variables are associated with the exposure (relevance assumption); that they are not associated with confounding factors (independence assumption); and that they affect the outcome only through their effects on the exposure of interest (exclusion restriction assumption).^12,13^ In addition to the biological relevance of proteins, the use of genetic variants associated with protein level (protein quantitative trait loci) as instrumental variables in MR has desirable properties in relation to these assumptions^14^ (Figure 1, Table S1). Protein quantitative trait loci variants are frequently derived from genomewide association studies (GWAS) using population-based genetic and circulating protein level data,^15,16^ fulfilling the relevance assumption by definition. The selection of genetic instruments mapping to the vicinity of the transcriptional gene unit (*cis*-acting variants), as opposed to those located more remotely (*trans*-acting variants), limits the scope for violating the exclusion restriction assumption, because protein quantitative trait loci variant effects on the outcome are likely mediated through expressions of the protein under consideration (no horizontal pleiotropy).^17^ Last, on the basis of the central dogma of molecular biology, it is implausible that *cis* variant instruments for protein exposures are conditional on the disease outcome, and therefore, it is reasonable to assume that protein traits are upstream of the disease outcome in any causal model. Throughout the article, we refer to this technique as *cis*-MR, which has been demonstrated to be able to predict efficacy of known drug targets for coronary heart disease.^14^

**Figure 1.**
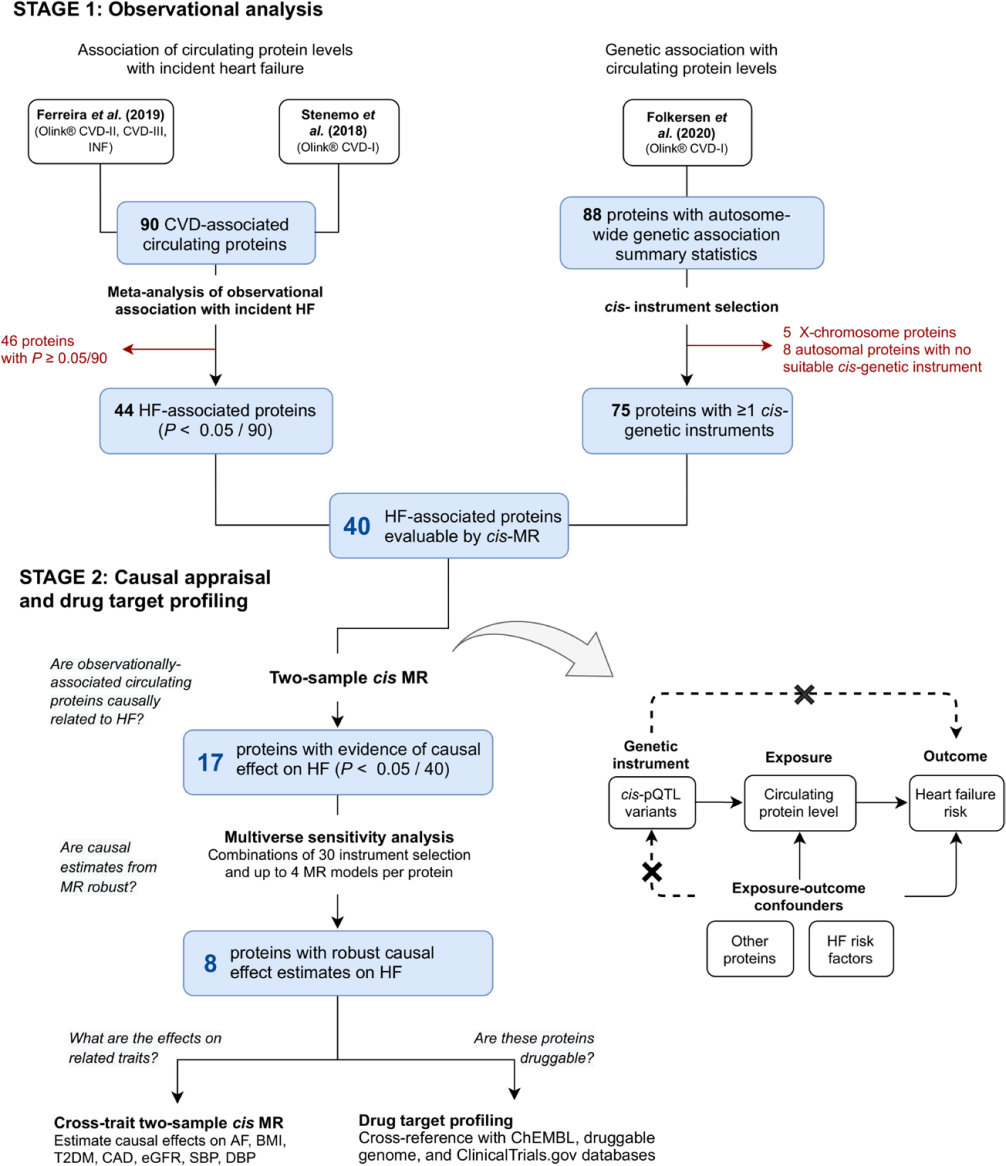
**A flow chart of the study design and a schematic illustration of *cis*-MR.** AF indicates atrial fibrillation; BMI, body mass index; CAD, coronary artery disease; *cis*-MR, Mendelian randomization using cis-acting protein quantitative trait loci instruments; DBP, diastolic blood pressure; eGFR, glomerular filtration rate; GWAS, genome-wide association study; HF, heart failure; LD, linkage disequilibrium; MR, Mendelian randomization; pQTL, protein quantitative trait loci; SBP, systolic blood pressure; and T2DM, type 2 diabetes mellitus.

Here, we report an integrated observational and *cis*-MR analyses of circulating protein levels for therapeutic target identification and prioritization in HF, focusing on up to 90 cardiovascular disease–related circulating proteins measured with the Olink Cardiovascular I circulating protein biomarker panel (Olink CVD-1) multiplexed affinity-based proximity extension assay^18^ (Figure 1). We perform meta-analysis of observational associations between circulating protein levels with incident HF^8,9^ estimated from 4 independent samples. We estimate the causality of these associations with *cis*-MR analysis by leveraging summary-level data from large GWASs of circulating levels of proteins under study^15^ and HF risk.^19^ We identify several likely causal proteins, report the anticipated effects on HF-related traits estimated through cross-trait *cis*-MR analysis, and characterize the druggability properties of these proteins as potential therapeutic targets for HF.

## Methods

### Data and Code Availability

For purposes of reproducing the results or replicating the procedure, the data and analysis code used in the main analysis have been made available to other researchers at https://github.com/alhenry/cvd1-hf. Other supporting data are available in the article, supplemental files, and referenced public datasets.

### Circulating Protein Level Measurement

Circulating protein levels were assessed using Olink Proseek Multiplex proximity extension assay^7,18^ technology and were quantified in a normalized protein expression unit, where 1 U difference represents a doubling of protein concentration.^20^ The present study focused on cardiovascular-disease related proteins available on the Olink CVD-1 panel, for which both observational associations with HF and genetic association estimates for *cis*-MR analysis were uniquely available at the time of the study. Observational association estimates with incident HF were available for 90 proteins reported in Ferreira et al^9^ and Stenemo et al,^8^ of which 88 had autosomewide genetic association results reported in Folkersen et al.^15^ In the observational studies, protein measures were taken at baseline. A detailed description of the methods used for protein quantification and the proteins measured by each of the included studies is provided in the Supplemental Methods, Table S2, and Figure 1.

### Study Population for Observational Analysis

We meta-analyzed observational association estimates between circulating protein level and incident HF from 4 independent samples reported in Ferreira et al^9^ and Stenemo et al^8^: HOMAGE (Heart Omics in Ageing)^21^ discovery, HOMAGE validation, PIVUS (Prospective Investigation of the Vasculature in Uppsala Seniors),^22^ and ULSAM (Uppsala Longitudinal Study of Adult Men).^23^ The HOMAGE discovery and validation samples were derived from 2 population cohorts and 1 clinical trial population: Health ABC (Health Aging and Body Composition),^24^ PREDICTOR (Valutazione della Prevalenza di Disfunzione Cardiaca Asintomatica e di Scompenso Cardiaco),^25,26^ and PROSPER (Prospective Study of Pravastatin in the Elderly at Risk).^27–29^ Individuals with prevalent HF at enrollment were excluded from the analysis. Incident HF was defined as the first diagnosis of HF, ascertained on the basis of hospital record review by trained physicians. The combined sample was composed of 3019 individuals (median age ranged from 70 to 78 years), among whom 732 incident HF events were observed during follow-up (median follow-up time ranged from 1.8 to 10 years). The studies were not able to differentiate between HF with reduced and preserved ejection fraction because of a lack of data on left ventricular ejection fraction. Characteristics of included studies are provided in Table 1 and the Supplemental Methods and in previous reports.^8,9^

**Table 1.**
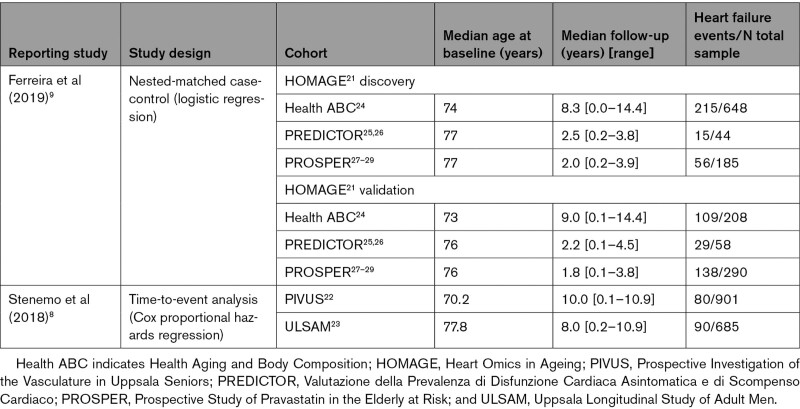
Summary of Study Characteristics Included in the Observational Meta-Analysis

### Statistical Analysis

#### Meta-Analysis of Observational Associations

We performed a fixed-effect meta-analysis using effect estimates from (1) HOMAGE discovery, (2) HOMAGE replication, (3) PIVUS, and (4) ULSAM. Effect estimates for HOMAGE discovery and HOMAGE replication were extracted from odds ratios calculated using multivariable logistic regression adjusting for age, sex, cohort, and follow-up time—which were used as matching variables in a matched, nested case-control design.^9^ For PIVUS and ULSAM, effect estimates were taken from hazard ratios calculated using Cox proportional hazard regression adjusting for age and sex.^8^ Hazard ratios and odds ratios were assumed to approximate to an equivalent risk ratio (RR), given that the outcome is rare.^30^ To make results comparable across studies and proteins, study-level circulating protein measures in the normalized protein expression unit are standardized by setting the mean to 0 and SD to 1 before running regression models, with an assumption that the SDs of circulating protein levels are similar across studies. To account for multiple testing, we implemented a Bonferroni-corrected allowable type I error rate (α) of 0.05/90 (number of proteins under study).

#### MR Analysis

We assessed the causality of associations for proteins that survived multiple testing correction in the observational analysis by performing 2-sample *cis*-MR using estimates of genetic association with circulating protein levels under study and with HF. Genetic associations with circulating protein levels were extracted from a GWAS meta-analysis of 14 cohorts composed of 30 931 subjects of European ancestry included in the SCALLOP consortium (Systematic and Combined Analysis of Olink Proteins).^15^ Genetic associations with HF were extracted from a GWAS meta-analysis of 47 309 all-cause HF cases from 26 studies of European ancestries included in the HERMES consortium (Heart Failure Molecular Epidemiology for Therapeutic Targets).^19^ Details of participating studies in each GWAS meta-analysis are provided in Tables S3 and S4.

Genetic instruments for proteins were selected from all biallelic single-nucleotide polymorphism available in both protein and outcome GWAS summary statistics with minor allele frequency >0.01 and located within 200 kbp upstream or downstream of the cognate protein-encoding transcription start and stop sites. Given that a gene *cis*- region constitutes only a small proportion of the genome, we relaxed the conventional genomewide significance *P* value threshold for instrument selection to *P*<1×10^–4^. To allow for an increased statistical power to detect an association, we implemented a relaxed linkage disequilibrium (LD) *r*^2^ threshold 0.4 and used MR models accounting for residual correlation.^31^ This threshold was based on a simulation study finding that unstable estimates caused by multicollinearity started to occur at a threshold correlation of around *r*^2^=0.36.^32^ Using these thresholds, we performed variant clumping implemented in PLINK 1.9^33^ to select *cis*- genetic instruments for each protein, with an LD model derived from individual-level genotype data imputed against the Haplotype Reference Consortium^34^ reference panel from a random sample of 10,000 UK Biobank^35^ participants.

MR estimates were calculated using the Wald ratio estimator for proteins with a single instrument selected, or the inverse-variance weighted (IVW) estimator for proteins with 2 or more instruments. The Wald ratio estimates are calculated as the regression coefficient for genetic association with the outcome divided by the regression coefficient for genetic association with circulating protein levels. The IVW estimates are calculated as the average of instrument ratio coefficients weighted by the inverse variance. Both estimates from observational association and MR analyses approximate a RR of HF per 1 SD increase in normalized protein expression unit (equivalent to per SD per doubling circulating protein concentration).

### Multiverse Sensitivity Analysis for MR

To test the robustness of estimates from the primary MR analysis, proteins with MR estimates surviving multiple testing correction (*P* value <0.05/numbers of observationally associated proteins with at least 1 instrument) were taken forward to undergo an in-depth, multiverse sensitivity analysis^36^ in which the stability of the effect estimates was evaluated under a wide combinations of instrument selection parameters and MR models. Thresholds for instrument selection (*P* value and *r*^2^) and alternative MR models were prioritized more than other possible parameters, such as LD reference population and genomic distance, because these parameters were observed to have the greatest influence on estimate stability in a previous systematic evaluation of methods for drug target MR.^14^ For each MR model, we computed causal estimates for all possible combinations of 5 LD *r^2^* thresholds (0.05, 0.1, 0.2, 0.4, and 0.6) and 6 *P* value thresholds (5×10^–8^, 1×10^–^^5^, 1×10^–^^4^, 1×10^–^^3^, 1×10^–^^2^, and 1/no threshold). These combinations included the parameters used in the primary MR analysis above and stringent parameters commonly used in conventional MR analysis of complex trait exposures.^37^ For proteins with a single *cis* instrument, the Wald ratio was the only model that could be tested; where 2 or more instruments were available, estimates were calculated with the IVW estimator and MR models using principal components^32^ with 90% variance and 99% variance explained; and where there were 3 or more instruments, we in addition calculated estimates using MR with Egger regression estimator^12^ (Figure S1). MR with principal components is an alternative model to account for correlation between instruments,^32^ and MR with Egger regression estimator provides estimates accounting for residual horizontal pleiotropy.^12^ To reduce spurious associations that may arise because of excess multicollinearity or bias toward the null because of weak instruments in 2-sample MR,^14^ outlier point estimates with a value outside 1.5 times the interquartile range above the upper quartile and below the lower quartile were removed. An association was declared as robust if all point estimates from the multiverse sensitivity analysis were directionally concordant with estimates from the primary MR analysis, including those on the basis of strict instrument selection parameters and a standard IVW model.

The IVW and MR with Egger regression estimates were calculated using the *MendelianRandomization* package in R,^38^ with a fixed-effect model for 3 or fewer genetic instruments, or a multiplicative random-effects model otherwise. To minimize erroneously low *P* value caused by a multicollinearity issue, correlation between instruments was accounted for by incorporating the instrument pairwise LD correlation matrix in the IVW and MR with Egger regression estimator models.^14,31^ The MR method with principal components was implemented using sample codes from the original publication.^32^ Genomic coordinates for all relevant analyses were based on Ensembl *GRCh37* reference.^39^

### Cross-Trait MR Analysis

To investigate the potential mechanisms through which candidate target proteins may influence HF risk, we performed an exploratory cross-trait MR to estimate the causal association of genetically predicted circulating protein levels with common risk factors and comorbidities of HF: coronary artery disease (CAD), atrial fibrillation, estimated glomerular filtration rate (eGFR), systolic blood pressure, diastolic blood pressure, type 2 diabetes, and body mass index. MR analysis was performed with the primary instrument selection strategy and MR model described in the MR Analysis section using publicly available GWAS statistics for the relevant traits (Table S5).^40–45^ To allow comparison across protein-trait pairs, effect estimates were converted to *Z* scores, calculated as log odd ratios divided by their SEs. The protein-trait MR association was considered potentially causal if the *P* value from the MR analysis was less than a conservative Bonferroni adjusted threshold of 0.05 divided by the number of protein-trait pairs.

### Evaluation of Druggability and Clinical Development Activity

We extracted the druggability profile of candidate target proteins from an updated list of druggable genes.^6^ To evaluate clinical development activity of candidate drugs targeting the candidate proteins, we queried the ChEMBL^46^ (release 27) database to get information on drug molecule types, approved indications, and target outcomes in clinical trials. We complemented this query by performing a manual search through the https://www.ClinicalTrials.gov website for each candidate target.

### Ethical Statement

All included studies were ethically approved by local institutional review boards, and all participants provided written informed consent. The analysis was conducted in accordance with guidelines for study procedures provided by the University College London Research Ethics Committee.

## Results

### Meta-Analysis of Observational Studies Reveals 44 Circulating Proteins Associated With Incident HF

Through a meta-analysis of observational associations from 4 independent samples, composing up to 732 incident HF events in 3019 subjects, we found 44 out of the 90 proteins were associated with incident HF after multiple testing adjustment at *P*<6.0×10^–4^ (α=0.05/90 proteins), including 22 associations that were not reported in the individual participating studies.^8,9^ Increasing circulating levels of all the 44 observationally associated proteins showed a risk-increasing effect on incident HF, with a median RR of 1.33 (interquartile range, 1.26–1.46). The largest effect sizes were observed in BNP (B-type natriuretic peptide; RR, 1.92 [95% CI, 1.70–2.18]) and NT-proBNP (N-terminal pro-BNP; RR, 1.85 [95% CI, 1.63–2.10]), 2 biomarkers that have been routinely used in the clinic to diagnose HF. We found no evidence of heterogeneity of the effect estimates after adjustment for multiple testing (*P*_heterogeneity_<0.05/44). Full study-level and meta-analysis estimates are provided in Table S6.

### Causal Effect Estimation With *cis*-MR

Of the 90 proteins being studied, *cis* region genetic association summary statistics were available for 83 proteins encoded by autosomal genes (Table S2). *Cis* region sizes varied according to gene length from 401 to 705 kbp and contained a mean of 1181 variants (SD, 498). Using the primary instrument selection parameter with LD *r*^2^ threshold of 0.4 and *P* value threshold of 10^–4^, we identified 75 proteins with 1 to 125 (median, 23) *cis*- genetic instrument, including 40 of the 44 observationally associated proteins. For comparison, conventional instrument selection parameters (LD *r*^2^<0.05, *P*<5×10^–8^) identified 70 proteins with 1 to 28 (median, 5) *cis*- genetic instruments. Instrument-specific estimates are provided in the data and code at https://github.com/alhenry/cvd1-hf/tree/main/resources.

**Figure 2.**
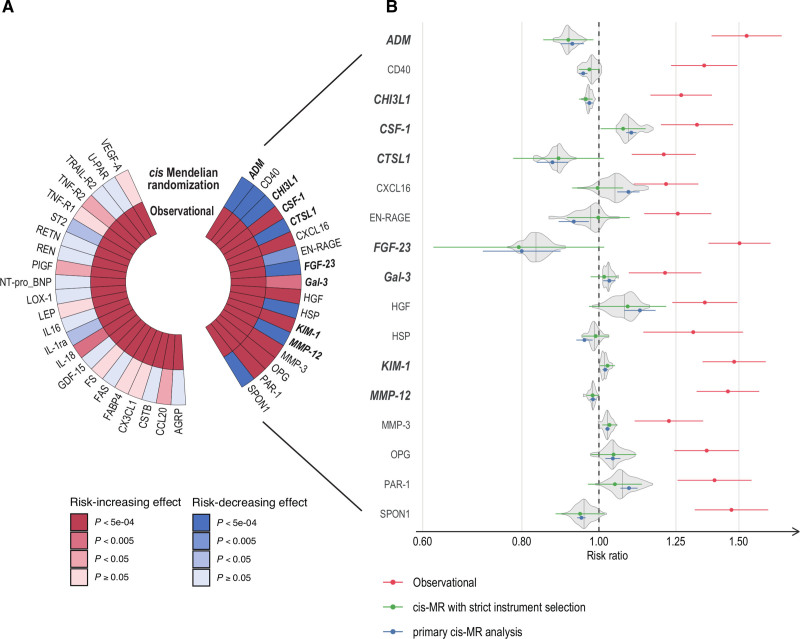
**Observational and MR estimates of protein–heart failure association. A**, Circular heatmap of association from 40 proteins associated with incident heart failure in observational studies *(P*<0.05/83=0.0006). The 2 circular lanes refer to results from 2 analyses: (1) observational analysis and (2) *cis*-MRwith partially correlated instruments. Color represents direction of effect and strength of association with heart failure measured by *P* value. **B**, Forest plot of risk ratio (hazard ratio from observational analysis and odds ratio from MR analysis) from 17 proteins associated with heart failure in MR analysis (*P*<0.05/40=0.001). Colored dots and error bars indicate the point estimate and 95% CIs. The gray violin plots around the MR estimates illustrate the distributions of odds ratio point estimates estimated from combinations of up to 30 instrument selection parameters and 4 MR models in multiverse sensitivity analysis, with medians of distribution shown as vertical lines within the violin plot. Proteins with consistent direction of effect as indicated by multiverse sensitivity analysis are highlighted in bold and italic font. Full protein names are provided in Table S2. *cis*-MR indicates Mendelian randomization using *cis*-acting protein quantitative trait loci instruments; and MR, Mendelian randomization.

The primary MR analysis suggested causal relationships for 17 of the 40 (43%) observationally associated proteins (*P*<0.05/40). The direction of effects for 16 of 17 proteins were consistent with those calculated using conventional MR parameters; however, only CHI3L1 survived the multiple testing correction (Figure 2). We also investigated the remaining 35 proteins that did not have an observational association with HF and with at least 1 *cis*- genetic instrument. Of these, we found an additional 9 proteins (26%) with evidence suggestive of a causal association with HF in MR (*P*<0.05/35). Full MR results are provided in Table S7.

### Multiverse Sensitivity Analysis Demonstrates Robust Causal Estimates for 8 HF-Associated Proteins

Noting that MR estimates are highly sensitive to choice of parameters for instrument and model selection,^17,47^ we tested the stability of the association estimates for each of the 17 HF-associated proteins for which the primary MR analysis suggested underlying causal effects, using a multiverse sensitivity analysis. We tested up to 120 combinations of commonly used parameters for instrument selection and MR models per protein, focusing on parameters that explain the largest variability in MR estimates on the basis of previous simulation and empirical studies,^14,32^ resulting in a total of 1850 individual effect estimates. We evaluated the distribution of the point estimates generated and compared these with the primary *cis*-MR analysis estimates and with estimates from conventional instrument selection parameters (Figure 2b, Table S8). For all 17 proteins under analysis, estimates from the primary *cis*-MR analysis were directionally concordant with median values of the multiverse analysis point estimate distributions and showed overlapping 95% CIs with estimates from *cis*-MR using conventional strict instrument selection parameters.

**Figure 3.**
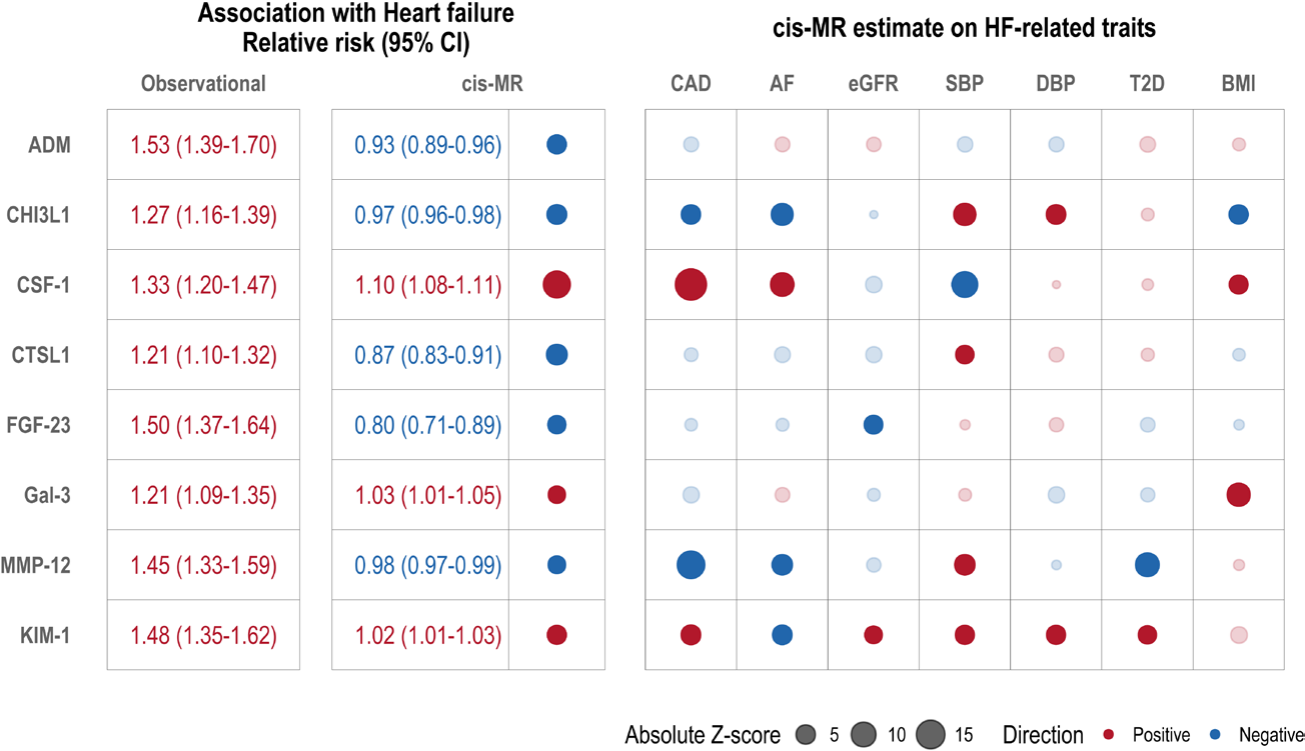
**Estimated effect of prioritized circulating protein levels with HF and related traits. Left**, Approximate relative risks of HF per doubling circulating protein levels as estimated with meta-analysis of observational data and *cis*-MR. **Right**, A matrix of estimated causal effect size of prioritized circulating protein levels (rows) on HF and related traits (columns) from cis-MR analysis as represented by bullet points. The size of the bullet represents the magnitude of estimated causal effect measured in absolute *Z* score. Bullet points with a darker shade indicate associations that survived multiple testing at *P* value <0.0009 (α=0.05/[8 proteins*7 traits, excluding HF]). Red indicates a risk/trait-increasing effect, and blue indicates a risk/trait-decreasing effect. ADM indicates adrenomedullin; AF‚ atrial fibrillation; BMI, body mass index; CAD, coronary artery disease; CHI3L1‚ chitinase 3-like 1; CSF-1‚ colony stimulating factor 1; CTSL1‚ cathepsin L; DBP, diastolic blood pressure; eGFR, estimated glomerular filtration rate; FGF-23‚ fibroblast growth factor 23; Gal-3‚ galectin-3; HF, heart failure; KIM-1‚ kidney injury molecule 1; MMP-12‚ matrix metallopeptidase 12; MR, Mendelian randomization; SBP, systolic blood pressure; and T2D, type 2 diabetes.

Furthermore, we identified robust evidence of a causal association with HF as indicated by sign concordance of all MR point estimates from the multiverse sensitivity analysis for 8 proteins: ADM (adrenomedullin), CHI3L1 (chitinase-3-like protein 1), CSF-1 (macrophage colony-stimulating factor 1), CTSL1 (cathepsin L1), FGF-23 (fibroblast growth factor 23), Gal-3 (galectin-3), MMP-12 (matrix metalloproteinase-12), and KIM-1 (kidney injury molecule 1). Increasing circulating levels of all 8 proteins were positively associated with risk of incident HF in the observational analysis. In the MR analysis, however, only 3 proteins (CSF-1, Gal-3, and KIM-1) showed positive associations with risk of HF, whereas the remaining 5 (ADM, CHI3L1, CTSL1, FGF-23, and MMP-12) showed negative associations, suggesting causal protective effects (Figure 3).

### Cross-Trait MR Analysis for Candidate Therapeutic Targets for HF

We took forward the 8 proteins robustly associated with HF and explored their causal effects on 7 HF-related traits (CAD, atrial fibrillation, estimated glomerular filtration rate, systolic blood pressure, diastolic blood pressure, type 2 diabetes, and body mass index), using the primary *cis*-MR analysis method (Figure 3). Of the 8 candidate proteins, 1 (ADM) was not associated with any trait other than HF, whereas the remaining 7 were associated with at least 1 other trait after multiple testing correction (*P*<0.05/8 proteins/7 traits excluding HF). Consistent with evidence from overexpression perturbation studies in animal models, Gal-3^48^ and CSF-1^49^ were positively associated with body mass index, a biomarker of adiposity and a known risk factor for HF.^50^ CHI3L1 and CTSL1 were protective for CAD, consistent with reports of cardioprotective effects in animal models of cardiac ischemia.^51,52^ A higher circulating CSF-1 level was associated with an increased risk of CAD,^53^ whereas MMP-12 showed a protective effect, consistent with previous reports.^16^ A higher level of FGF-23 was associated with a lower estimated glomerular filtration rate, consistent with findings from preclinical models in which FGF-23 deficiency was associated with worsening renal failure and cardiac hypertrophy.^54^

### Appraisal of Druggability and Existing Approved or Clinical-Phase Drug Candidates for Candidate Protein Targets

To evaluate the druggability and drug development activities of candidate targets, we searched through a list of druggable genes,^6^ the ChEMBL (release 27) drug discovery database, and a clinical trial registry (https://www.clinicaltrials.gov, accessed on December 1, 2020). We grouped candidate targets into 3 categories corresponding to the highest status in the drug development pipeline: *approved* (targeted by drugs already approved for 1 or more conditions), *in development* (currently being investigated in clinical trials), and *druggable* (listed as druggable targets; Table 2). A candidate drug targeting adrenomedullin, adrecizumab (a humanized, monoclonal, nonneutralizing antibody against the N terminus of ADM^55^), is entering phase II trials for septic shock (URL: https://www.ClinicalTrials.gov; Unique identifier: NCT03085758), cardiogenic shock (Unique identifier: NCT03989531), and acute HF (Unique identifier: NCT04252937). A modified citrus pectin Gal-3 inhibitor has been evaluated for effects markers of collagen metabolism in patients with hypertension in a proof-of-concept clinical trial for cardiac fibrosis.^56^ CSF-1 and MMP-12 inhibitors are currently being evaluated in clinical trials for non-HF conditions. Burosumab, a monoclonal antibody FGF-23 inhibitor, has already been approved for treating X-linked hypophosphatemia and hypophosphatemic rickets. Although we found no ongoing trials specific for CHI3L1 or CTSL1, inhibition of CTSL1 is proposed as potential treatment for SARS-CoV-2 infection, and several approved agents show inhibitory activity against CTSL1.^57^ With the exception of KIM-1, all 7 other proteins are predicted to be secreted in at least 1 tissue according to the Human Protein Atlas database.^58^ KIM-1 is also not currently listed as a potential drug target according to the druggable gene list, ChEMBL release 27, and https://www.ClinicalTrials.gov databases.

**Table 2.**
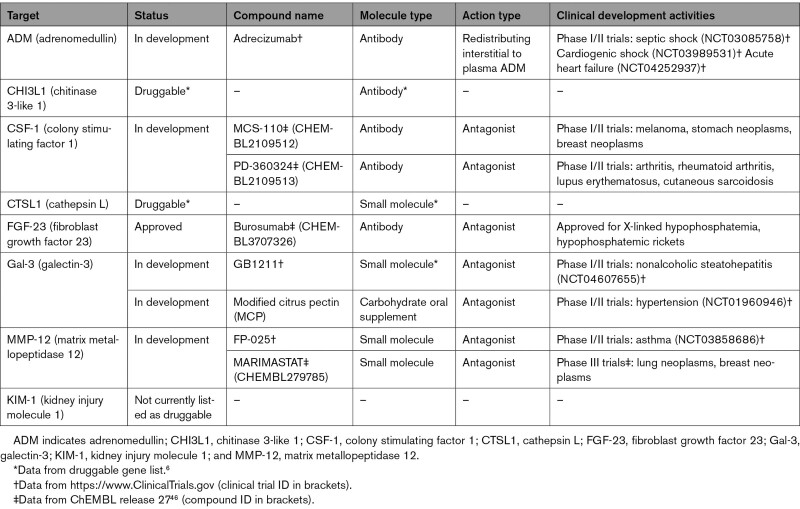
Summary of Druggability and Clinical Development Activity for Heart Failure Associated With Causal Associations on Mendelian Randomization Analysis

## Discussion

### Principal Findings

We investigated 90 circulating proteins for their association with incident HF in a population of 3019 individuals with 732 events. A total of 44 proteins had positive associations with risk of incident HF, 22 of which were not reported in the participating studies. These included associations with incident HF reported elsewhere such as Gal-3, HGF, and Resistin,^59–61^ proteins such as CXCL16 with reported associations with prognosis in HF,^62^ and with cardiac fibrosis on cardiac magnetic resonance imaging in HF including MMP3.^63^ Among the novel associations to highlight, CTSL1 is a potent endoprotease linked to the development of dilated cardiomyopathy and HF in mouse models.^64,65^ We used *cis*-MR to estimate whether the observational protein-HF associations reflected an underlying causal relationship. Of the 40 proteins for which *cis* genetic instruments were available, 17 showed evidence suggestive of causal effects, of which 8 were robust to multiverse sensitivity analysis. Among these 8 HF-associated proteins, 3 were positively associated with risk of HF (CSF-1, Gal-3, and KIM-1), and 5 were negatively associated, consistent with causally protective effects (ADM, CHI3L1, CTSL1, FGF-23, and MMP-12). Seven are known or predicted to be druggable by conventional therapeutic modalities, and therapeutic agents targeting 2 of the identified proteins are currently under evaluation in phase II clinical trials: adrecizumab, an ADM agonist, for acute HF and cardiogenic shock,^55^ and modified citrus pectin, a Gal-3 antagonist, for cardiac fibrosis.^56^ We note that CTSL1 inhibition has been proposed as a potential treatment for COVID-19^66^; our results signal HF as a potential safety liability of this therapeutic approach. Our findings provide evidence supporting the therapeutic hypotheses underpinning 2 drug development programs for HF and more broadly highlight the emerging opportunities to explore human causal biology of complex disease using population-scale genomic and proteomic data.

### Concordance of Observational and Causal Associations for Identified Proteins

One of the key strengths of study is the triangulation of evidence between observational and MR analyses for a consistently measured set of cardiovascular proteins. For all the protein-HF associations that were identified in our meta-analysis, there was a positive association, ie, a higher protein concentration was associated with an increased risk of incident HF. This is consistent with previously reported biomarker association studies with incident HF; for example, a study of incident HF in the Framingham population identified 18 associated circulating biomarkers, of which 17 were positive associations.^67^ When we estimated the causal association of the observationally associated HF proteins, however, we found that the observational and causal association estimates were frequently discordant with opposing direction of effects. For example, 5 proteins with an estimated causally protective effect were found to have a positive association with incident HF, including MMP-12 and ADM. In the case of MMP-12, our findings are consistent with previous reports on the associations between MMP-12 and CAD.^16,68^ These discordant findings may be explained by subclinical or predisease leading to higher levels of these proteins that precedes the clinical diagnosis of HF, potentially as an adaptive feedback response to mitigate the disease process. The median baseline age in the included studies ranged from 70 to 78 years, and it is likely that subclinical alterations in cardiac structure and function occurred before incident HF, which was defined as the first HF hospitalization. Concordant observational and causal associations (CSF-1, Gal-3, and KIM-1) may be explained either by upstream processes driving risk or by reverse causation where a positive feedback loop exists between the HF and expression of the protein. For several proteins, including established clinical biomarkers NT-proBNP and ST2, we found positive observational associations but were unable to detect causal effects by MR analysis. In these, the observational associations may be interpreted as noncausal, arising from reverse causation. We cannot, however, exclude a type 2 error caused by imprecision of the MR estimates.

### Comparison With Other Studies

To our knowledge, our study represents the first large-scale analysis of incident HF that combines observational associations of circulating proteins with a systematic appraisal of causal effects using MR. Our results were consistent with previously reported findings from MR studies of NT-proBNP and GDF-15, which did not report evidence of a strong causal relationship between these proteins and risk of HF.^69,70^ Our approach of triangulating evidence from observational association and MR represents a pragmatic approach to screen and prioritize targets for therapeutic development, according to the relative strength of evidence from analysis of the data available.^71^ In our study, we used a method for *cis*-MR that incorporates the LD correlation structure within the causal model and provides estimates with higher precision.^31^ We combined this primary approach with a new technique to evaluate the robustness of the identified protein-HF associations that involved systematically testing multiple combinations of model parameter selection in a multiverse sensitivity analysis, enabling us to deprioritize proteins with unstable estimates. Using this framework, we found evidence supporting a causal relationship for 8 of the 40 HF-associated proteins tested, compared with a single association for CHI3L1 that was identified using conventional approaches. For example, the estimates for CTSL1 and FGF-23 generated with this approach more clearly suggest a causal effect compared with those on the basis of more stringent instrument selection (Figure 2b, Table S7).

### Implications for Therapeutics Targeting HF

All 8 proteins with estimated causal effects, except ADM, were associated with HF-related traits in an exploratory *cis*-MR cross-trait analysis, including upstream HF risk factors. Distinct pathobiological pathways and proteomic signatures are described for subgroups of patients with HF, such as those defined by left ventricular ejection fraction^72^; however, we were unable to perform a stratified analysis because of the limited phenotype data available at the time of HF diagnosis. To leverage the full potential of proteomics and genomics in understanding HF and identifying drug targets, there is a need to decompose HF into phenotypic components, including those of cardiac dysfunction and fluid congestion, which characterize this condition. ADM and CTSL1 are notable among our findings because their protective effect against the risk of HF was not explained by association with upstream risk factor traits. ADM is a circulating peptide hormone synthesized by endothelial and vascular smooth muscle cells, the biologically active form of which has been proposed as a marker and inhibitor of tissue fluid congestion, a hallmark feature of HF.^55^ Consistent with our results, it has been hypothesized that ADM may play a protective role in HF development and progression by maintaining vascular integrity, inducing vasodilatation, and inhibiting the renin-angiotensin-aldosterone system.^55^

### Limitations

Although the clinical ascertainment of HF was consistent across the studies included in the observational analysis and in HF GWAS, the interpretation of our findings is limited by the lack of detailed phenotyping by pathogenesis and phenotypes of cardiac structure and function. Our MR framework, including the prioritization of parameters for the multiverse analysis, was based on previous studies of gene transcript exposures which demonstrated robust and reproducible MR estimates^73^; however the scope of our multiverse analysis was limited by the computational burden inherent in the approach. There is a lack of consensus about the optimal approach to *cis*-MR, and we were unable to empirically replicate our findings in an independent sample because none were available at the time of the study. It is possible for proteins with an important causal contribution to HF risk to have a null observational association in this study because of negative confounding or imprecision of the estimates. Given that circulating protein concentrations are measured in a relative normalized protein expression unit,^20^ the derived effect estimates are rarely representative of the absolute magnitude of effect on HF and are not directly comparable across proteins. The expected causal direction of effects, however, can inform potential efficacy and on-target side effects, which can be formally investigated further in clinical trials. Further studies are needed to corroborate and extend our findings, to include a larger number of protein biomarkers, and to explore the relationship of the identified proteins with disease subtypes. These studies will be enabled by the rapidly increasing availability of proteomic and genomic information in large populations from large health care–linked biobanks.

## Conclusions

In conclusion, we evaluated 90 cardiovascular-related proteins through observational and MR analysis using population-based proteomic data and identified 7 candidate drug targets for HF. Of these, 2 proteins (ADM and Gal-3) are currently under evaluation in clinical trials for HF, and 5 (CHI3L1, CSF-1, CTSL1, FGF-23, and MMP-12) represent novel putative therapeutic targets for HF. This study provides an example of the opportunities for human target prioritization that are enabled by emerging population-based genomic and proteomic data resources. Proteomewide studies incorporating both direct association with target outcomes and genetic-based inference through MR are likely to provide important new tools for therapeutic target discovery and prioritization.

## Article Information

### Acknowledgments

The authors thank the participants of PIVUS, ULSAM, and studies included in the HOMAGE, SCALLOP, and HERMES consortia. The authors thank Olink Proteomics for providing proteomics assays used in PIVUS, ULSAM, and studies in the HOMAGE and SCALLOP consortia. The authors thank the investigators of consortia and research groups who performed GWAS analyses and made summary statistics data available to use in the present study. The authors acknowledge the use of the UCL Myriad High Performance Computing Facility (Myriad@UCL), and associated support services in the completion of this work. A.H., A.M., A.D.H.‚ and R.T.L. conceived and designed the study. J.S., L.L, J.Ä., and F.Z. provided data for observational meta-analysis. A.M. and R.T.L. provided data for MR analysis. A.H., M.G.-M.‚ and J.P.F. performed data analyses, with input from C.F., A.F.S., R.T.L., and A.D.H. A.H. and R.T.L. wrote the initial draft, which was further reviewed and edited by M.G.-M.‚ C.F., A.F.S, R.K., and A.D.H. A.M., A.D.H, and R.T.L. jointly supervised the work. All authors provided critical revisions in subsequent drafts and gave approval to the final version for submission. The corresponding author confirms that all listed authors meet authorship criteria and that no others meeting the criteria have been omitted.

### Sources of Funding

This work is supported by the BigData@Heart Consortium funded by the Innovative Medicines Initiative-2 Join Undertaking under grand agreement No. 116074, the UCL Hospitals NIHR Biomedical Research Centre, the British Heart Foundation (BHF) Research Accelerator Award (AA/18/6/34223), and UK Research and Innovation/ NIHR-funded Mulimorbidity Mechanism and Therapeutics Research Collaborative (MR/V033867/1). A.H. (FS/18/65/34186) and M.G.-M. (FS/17/70/33482) acknowledge support by the BHF Cardiovascular Biomedicine PhD studentship. A.D.H. is an NIHR Senior Investigator. A.F.S is supported by BHF grant PG/18/503383, and acknowledges support by grant R01 LM010098 from the National Institutes of Health (United States). R.K. acknowledges support by the Walker-Inman Endowment, the Mandel Foundation, and grant R03 HL144812-01. L.L. is supported by a grant from the Swedish Heart-Lung Foundation. J.Ä. is supported by grants from the Swedish Research Council (2019-01015 and 2020-00243) and the Swedish Heart-Lung Foundation (20180343 and 20210357). R.T.L. is supported by a UK Research and Innovation Rutherford Fellowship hosted by Health Data Research UK (MR/S003754/1) and BigData@Heart Consortium funded by the Innovative Medicines Initiative-2 Joint Undertaking under grant agreement No. 116074. C.F., A.F.S., and R.T.L. acknowledge additional support from UCL Hospitals NIHR Biomedical Research Center.

### Disclosures

A.F.S. has received Servier funding for an unrelated work. The views expressed in this article are the personal views of M.G.-M. and do not represent the views of her current employer, the European Medicines Agency. J.P.F. consults for Boehringer Ingelheim and has received research support from Boehringer Ingelheim, Novartis, AstraZeneca, and Bayer through his institution. J.S. reports stock ownership in companies providing services to Amgen, AstraZeneca, Boehringer, Bayer, Eli Lilly, Itrim, Janssen, Novo Nordisk, Pfizer, and Takeda, outside the submitted work. J.Ä. has served on advisory boards for AstraZeneca and Boehringer Ingelheim and received lecturing fees from AstraZeneca and Novartis, all unrelated to the present project. The other authors report no conflicts.

### Supplemental Material

Supplemental Methods

Figure S1

Tables S1–S8

## Supplementary Material
