# Supplementary material for: Therapeutic Targets for Heart Failure Identified Using Proteomics and Mendelian Randomization
Source: Circulation. 2022 Mar 18;145(16):1205–17. doi: 10.1161/CIRCULATIONAHA.121.056663 (PMC9010023; doi:10.1161/CIRCULATIONAHA.121.056663)
Supplement: Supplementary file 1 [file cir-145-1205-s001.pdf]

# SUPPLEMENTAL MATERIALS

*Therapeutic targets for heart failure identified using proteomics and Mendelian randomization*

Albert Henry, MD, MSc; María Gordillo-Marañón, MSc; Chris Finan, PhD; Amand F Schmidt, MSc, PhD; João Pedro Ferreira, MD, PhD; Ravi Karra, MD, MHS; Johan Sundström, MD, PhD; Lars Lind, MD, PhD; Johan Ärnlöv, MD, PhD; Faiez Zannad, MD, PhD; Anders Mälarstig, MSc, PhD; Aroon D. Hingorani, MBBS, PhD, FRCP; R. Thomas Lumbers, MB BChir, PhD; HERMES and SCALLOP Consortia

# Table of contents

**Table of contents** **2**

**SUPPLEMENTAL METHODS** **3**

Cohort Descriptions 3

Circulating protein level measurement 4

**SUPPLEMENTAL FIGURE** **6**

**Supplemental Figure 1.** Schematic illustration of MR analysis in the present study 6

**SUPPLEMENTAL TABLES** **7**

**Supplemental Table 1.** Summary of key assumptions for valid Mendelian randomization (MR) in relation to protein trait exposures instrumented by cis- protein trait quantitative loci (pQTL) genetic variants 7

**Supplemental Table 2.** List of Olink CVD-1 proteins included in the present study 8

**Supplemental Table 3.** Studies included in the GWAS meta-analysis of circulating protein level from SCALLOP Consortium 11

**Supplemental Table 4.** Studies included in the GWAS meta-analysis of heart failure from HERMES Consortium 12

**Supplemental Table 5.** Summary of GWAS summary statistics used in the primary and cross-trait MR analyses 16

**Supplemental Table 6.** Study-level and meta-analysis estimates of observational associations between circulating protein levels and incident HF 17

**Supplemental Table 7.** Cis- Mendelian randomization (MR) analysis results 19

**Supplemental Table 8.** Multiverse sensitivity analysis results for 17 proteins with cis-MR associations which survived multiple testing correction 21

# SUPPLEMENTAL METHODS

## Cohort Descriptions

### HOMAGE

The Heart 'omics' in AGEing (HOMAGE) is an international collaborative project aiming to build a merged clinical database from population-based cohorts and clinical trials to study the role of biomarkers in the progression of heart failure.[21] The present study included three cohorts participated in the HOMAGE project with Olink protein biomarker data: Health Aging and Body Composition (Health ABC)[24], *Valutazione della PREvalenza di DIsfunzione Cardiaca asinTOmatica e di scompenso cardiaco* (PREDICTOR)[25,26], and Prospective Study of Pravastatin in the Elderly at Risk (PROSPER)[27–29]. For meta-analysis, individual-level data from the three cohorts were merged and further separated into discovery and validation samples. Details of the three cohorts are presented below.

- **Health ABC**

  Health ABC is a population-based study of 1584 women and 1491 men aged 70 to 79 years at enrollment (April 1997–June 1998), identified from a random sample of white Medicare beneficiaries and all age-eligible black residents in Pittsburg, Pennsylvania, and Memphis, Tennessee. Incident HF was defined as all first hospital admissions with an overnight stay related to HF as confirmed by a physician and a documented treatment for HF.[24]

- **PREDICTOR**

  PREDICTOR is a cross-sectional, population-based study of a random sample of 2001 residents aged 65-84 years old recruited between June 2007 and January 2010 from four cities in the Lazio, Central Italy region. HF diagnosis was made on the basis of clinical evaluation according to the 2005 ESC criteria, presence of signs and symptoms consistent with New York Heart Association class >1, and echocardiographic evidence of systolic or diastolic left ventricular dysfunction. Case status was confirmed by a panel of three cardiologists on the basis of a majority decision.[25,26]

- **PROSPER**

  PROSPER is a randomized, double-blind, placebo-controlled trial designed to test the hypothesis that pravastatin will diminish risk of subsequent major vascular events. Participants include 2000 women and 2804 men aged 70-82 years with a history of vascular disease or at high risk for developing vascular disease recruited between December 1997 and May 1999 from Scotland, Ireland, and the Netherlands. Patients with HF were excluded at baseline. Incident HF was defined as presence of a hospital admission record of HF diagnosed from a combination of symptoms and signs, chest radiograph, and echocardiograph.[27–29]

### PIVUS and ULSAM

PIVUS (Prospective Investigation of the Vasculature in Uppsala Seniors) is a population-based cohort which recruited 1,016 subjects aged 70 years living in the community of Uppsala, Sweden. Subjects were chosen from a community register and were invited in a randomized order, with a participation rate of 50.1%.[22] The ULSAM study included 838 men living in Uppsala at age 77 between 1998 and 2001.[23] In the analyses included in the present study, participants from the two cohorts were followed up to 11 years from baseline until heart failure diagnosis, death, or end of follow-up (10 June 2014 in PIVUS and 31 December 2008 in ULSAM). Diagnosis of heart failure was derived from medical records based on the presence of International Classification of Diseases, Ninth Revision (ICD-9) code 428, ICD-10 code I50, or ICD-10 I11 (hypertensive heart disease). Probable heart failure cases were reviewed by physicians blinded to baseline data and classified as definite, questionable, or miscoded according to the European Society of Cardiology definitions. All definite cases were included in the analysis.

### Circulating protein level measurement

Circulating protein levels were assessed using Olink Proseek Multiplex proximity extension assay (PEA)[9] technology, which simultaneously measures 92 human protein biomarkers.

Briefly, the PEA technology uses a pair of oligonucleotide-labeled antibodies which bind to the target protein in the sample. On target binding, the oligonucleotide sequences come in close proximity, hybridize, and then are extended by a DNA polymerase to form a polymerase chain reaction (PCR) amplicon. The resulting amplicon is detected and quantified using real-time quantitative PCR method to estimate the circulating protein abundance.[7,18] Protein levels are reported in NPX (normalized protein expression), a normalized unit in log2 scale calculated from cycle threshold (Ct) values, where 1 unit difference represents a doubling of protein concentration.[20]

The present study evaluated the observational associations of circulating proteins included in Olink CVD-1 panel with incident HF (including both HF with reduced and preserved ejection fraction) reported in either Stenemo et al. (2018)[8] or Ferreira *et al*. (2019)[9]. Of 92 proteins in the Olink CVD-1 panel, Stenemo et al. (2018) excluded 12 proteins with missingness ≥15% and imputed 22 proteins with values below the lower limit of detection to half the lower limit of detection.[8] Ferreira et al. (2019) evaluated 252 circulating protein levels (after exclusion of 15 proteins below the limit of detection) measured with Olink CVD-II, CVD-III, and INF panels, 83 of which are available in Olink CVD-1. Collectively, the two studies report association results of 90 out of 92 proteins in the CVD-I panel with incident HF (minus results for IL-4 and mAmp). Of these, 88 proteins had autosome-wide genetic association results reported in Folkersen et al. (2020)[15]. For the *cis*-Mendelian randomization analysis, 5 proteins encoded by non-autosomal genes were excluded, resulting in 83 proteins evaluated in the *cis*-MR analysis. More details about the CVD-1 proteins are available from the manufacturer webpage (https://www.olink.com/content/uploads/2015/12/0696-v1.3-Proseek-Multiplex-CVD-I-Validation-Data_final.pdf).

# SUPPLEMENTAL FIGURE

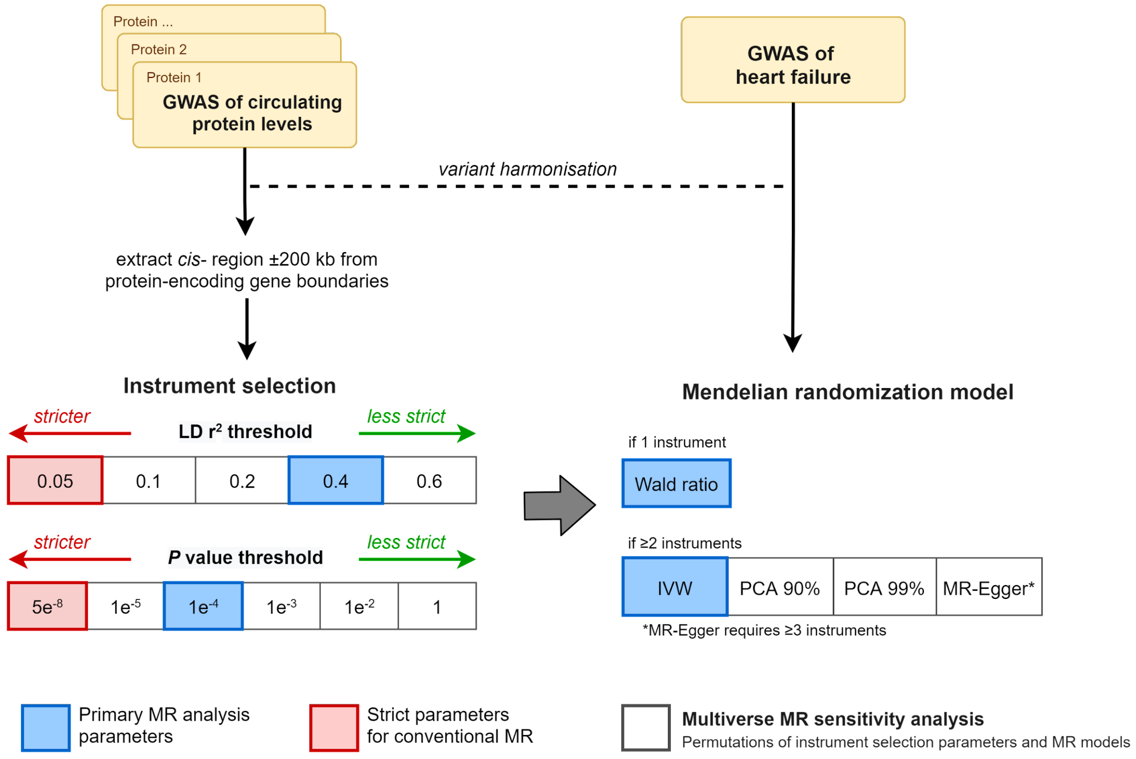


**Supplemental Figure 1.** Schematic illustration of MR analysis in the present study. MR, Mendelian randomization; LD, linkage disequilibrium; GWAS, genome-wide association study; IVW, inverse-variance weighted MR estimator; PCA 90%, principal component - based MR analysis with 90% variance explained; MR-Egger, Mendelian randomization with Egger regression estimator.

## SUPPLEMENTAL TABLES

**Supplemental Table 1.** Summary of key assumptions for valid Mendelian randomization (MR) in relation to protein trait exposures instrumented by *cis-* protein trait quantitative loci (pQTL) genetic variants

| MR assumption | Key strength of *cis-* pQTL instruments for protein exposures | Potential bias | Study design consideration to minimise bias |
|---|---|---|---|
| **Relevance:** *genetic variants are associated with the exposure of interest* | *cis-* pQTL variants derived from genome-wide association studies using circulating protein data are associated with expression of protein of interest by definition | • Spurious pQTL associations relating to assay binding (cross reactivity, epitope effects)<br>• Limited abundance of protein in plasma or limited assay affinity leading to suboptimal pQTL detection<br>• Limited contribution of cis-variant to plasma protein variance in population | • Use of largest available pQTL data for the set of proteins of interest from GWAS meta-analysis of circulating proteins<br>• Protein-level quality control based on limit of detection<br>• Instrument selection based on strength of association with circulating protein levels<br>• Use of multi-instrument MR model to improve statistical power |
| **Independence:** *genetic variants is not associated with confounders between exposure and outcome of interests* | It is unlikely that conventional confounding factors between protein and outcome (e.g. expression of other proteins, risk factors affecting both levels of protein and outcome of interest) can affect genetic variation | • Confounding by population structure<br>• High linkage disequilibrium between selected cis-pQTL instrument variants and coding or regulatory variants affecting the expression or function of other proteins<br>• Overlapping gene regions | • Adjustment for population structure<br>• Instrument pruning based on linkage disequilibrium metrics<br>• Instrument is selected from ±200 kb flanking region of protein-encoding genes to limit contamination by variants with structural gene |
| **Exclusion restriction:** *Genetic variants affect the outcome only through effects on exposure (no horizontal pleiotropy)* | The central dogma of molecular biology denotes that the functional form of protein is expressed through transcription of cognate gene and translation machinery, implicating that any effect of *cis*-pQTL variants on outcome is downstream of its effect on the protein of interest | • Alternatively spliced gene resulting in the expression of additional distinct proteins, other than the protein of interest, with effects on the outcome<br>• Selected *cis*-pQTL instruments affects the expression or function of a microRNA that regulates the translation of transcripts from multiple other genes | • Multiverse sensitivity analysis with permutation of instrument selection parameters and MR models to test robustness of the main MR estimates that might arise due to presence of invalid cis-pQTL instruments |

**Supplemental Table 2.** List of Olink CVD-1 proteins included in the present study

| Protein short name | Protein full name | UniProt ID | Encoding gene (HGNC) | CHR | Transcript start | Transcript end | strand | Association data availability | | |
|---|---|---|---|---|---|---|---|---|---|---|
| | | | | | | | | Ferreira 2019 | Stenemo 2018 | Folkersen 2020 (cis region) |
| ADM | Adrenomedullin | P35318 | *ADM* | 11 | 10326227 | 10328944 | 1 | ✓ | ✓ | ✓ |
| AGRP | Agouti-related protein | O00253 | *AGRP* | 16 | 67516474 | 67517716 | -1 | ✓ | ✓ | ✓ |
| Beta-NGF | Beta-nerve growth factor | P01138 | *NGF* | 1 | 115828539 | 115880857 | -1 | ✓ | | ✓ |
| BNP | Natriuretic peptides B | P16860 | *NPPB* | 1 | 11917521 | 11918988 | -1 | ✓ | | |
| CA-125 | Ovarian cancer-related tumor marker CA 125 | Q8WXI7 | *MUC16* | 19 | 8959520 | 9092018 | -1 | | ✓ | ✓ |
| CASP-8 | Caspase-8 | Q14790 | *CASP8* | 2 | 202098166 | 202152434 | 1 | ✓ | ✓ | ✓ |
| CCL20 | C-C motif chemokine 20 | P78556 | *CCL20* | 2 | 228678558 | 228682272 | 1 | ✓ | ✓ | ✓ |
| CCL3 | C-C motif chemokine 3 | P10147 | *CCL3* | 17 | 34415602 | 34417487 | -1 | ✓ | ✓ | ✓ |
| CCL4 | C-C motif chemokine 4 | P13236 | *CCL4* | 17 | 34430983 | 34433014 | 1 | ✓ | ✓ | ✓ |
| CD40 | Tumor necrosis factor receptor superfamily member 5 | P25942 | *CD40* | 20 | 44746911 | 44758502 | 1 | ✓ | ✓ | ✓ |
| CD40-L | CD40 ligand | P29965 | *CD40LG* | X | 135730352 | 135742549 | 1 | ✓ | ✓ | |
| CHI3L1 | Chitinase-3-like protein 1 | P36222 | *CHI3L1* | 1 | 203148059 | 203155877 | -1 | ✓ | ✓ | ✓ |
| CSF-1 | Macrophage colony-stimulating factor 1 | P09603 | *CSF1* | 1 | 110453233 | 110473614 | 1 | ✓ | ✓ | ✓ |
| CSTB | Cystatin-B | P04080 | *CSTB* | 21 | 45192393 | 45196326 | -1 | ✓ | | ✓ |
| CTSD | Cathepsin D | P07339 | *CTSD* | 11 | 1773982 | 1785222 | -1 | ✓ | ✓ | ✓ |
| CTSL1 | Cathepsin L1 | P07711 | *CTSL* | 9 | 90340434 | 90346307 | 1 | ✓ | ✓ | ✓ |
| CX3CL1 | Fractalkine | P78423 | *CX3CL1* | 16 | 57406370 | 57418960 | 1 | ✓ | ✓ | ✓ |
| CXCL1 | C-X-C motif chemokine 1 | P09341 | *CXCL1* | 4 | 74735121 | 74736959 | 1 | ✓ | ✓ | ✓ |
| CXCL16 | C-X-C motif chemokine 16 | Q9H2A7 | *CXCL16* | 17 | 4636821 | 4643217 | -1 | ✓ | ✓ | ✓ |
| CXCL6 | C-X-C motif chemokine 6 | P80162 | *CXCL6* | 4 | 74702214 | 74704477 | 1 | ✓ | ✓ | ✓ |
| Dkk-1 | Dickkopf-related protein 1 | O94907 | *DKK1* | 10 | 54074056 | 54077417 | 1 | ✓ | ✓ | ✓ |
| ECP | Eosinophil cationic protein | P12724 | *RNASE3* | 14 | 21359558 | 21360507 | 1 | | ✓ | ✓ |
| EGF | Epidermal growth factor | P01133 | *EGF* | 4 | 110834040 | 110933422 | 1 | | ✓ | ✓ |
| EN-RAGE | Protein S100-A12 | P80511 | *S100A12* | 1 | 153346184 | 153348125 | -1 | ✓ | ✓ | ✓ |
| ESM-1 | Endothelial cell-specific molecule 1 | Q9NQ30 | *ESM1* | 5 | 54273692 | 54281491 | -1 | | ✓ | ✓ |
| FABP4 | Fatty acid-binding protein, adipocyte | P15090 | *FABP4* | 8 | 82390654 | 82395498 | -1 | ✓ | ✓ | ✓ |
| FAS | Tumor necrosis factor receptor superfamily member 6 | P25445 | *FAS* | 10 | 90750414 | 90775542 | 1 | ✓ | ✓ | ✓ |
| FGF-23 | Fibroblast growth factor 23 | Q9GZV9 | *FGF23* | 12 | 4477393 | 4488894 | -1 | ✓ | ✓ | ✓ |
| FS | Follistatin | P19883 | *FST* | 5 | 52776239 | 52782964 | 1 | ✓ | ✓ | ✓ |
| GAL | Galanin peptides | P22466 | *GAL* | 11 | 68451943 | 68458643 | 1 | | ✓ | ✓ |
| Gal-3 | Galectin-3 | P17931 | *LGALS3* | 14 | 55595762 | 55612121 | 1 | ✓ | ✓ | ✓ |
| GDF-15 | Growth/differentiation factor 15 | Q99988 | *GDF15* | 19 | 18492980 | 18499986 | 1 | ✓ | ✓ | ✓ |

| | | | | | | | | | | |
|---|---|---|---|---|---|---|---|---|---|---|
| GH | Growth hormone | P01241 | *GH1* | 17 | 61994560 | 61996179 | -1 | ✓ | ✓ | ✓ |
| HB-EGF | Proheparin-binding EGF-like growth factor | Q99075 | *HBEGF* | 5 | 139712428 | 139726216 | -1 | ✓ | ✓ | ✓ |
| HGF | Hepatocyte growth factor | P14210 | *HGF* | 7 | 81328322 | 81399514 | -1 | ✓ | ✓ | ✓ |
| hK11 | Kallikrein-11 | Q9UBX7 | *KLK11* | 19 | 51525487 | 51531295 | -1 | | ✓ | ✓ |
| HSP | Heat shock 27 kDa protein | P04792 | *HSPB1* | 7 | 75931861 | 75933612 | 1 | ✓ | | ✓ |
| IL-18 | Interleukin-18 | Q14116 | *IL18* | 11 | 112013974 | 112034840 | -1 | ✓ | ✓ | ✓ |
| IL-1ra | Interleukin-1 receptor antagonist protein | P18510 | *IL1RN* | 2 | 113868693 | 113891593 | 1 | ✓ | ✓ | ✓ |
| IL-27 | Interleukin-27 | Q8NEV9 | *IL27* | 16 | 28510683 | 28518155 | -1 | ✓ | ✓ | ✓ |
| IL-6 | Interleukin-6 | P05231 | *IL6* | 7 | 22765503 | 22771621 | 1 | ✓ | ✓ | ✓ |
| IL-6RA | Interleukin-6 receptor subunit alpha | P08887 | *IL6R* | 1 | 154377669 | 154441926 | 1 | ✓ | ✓ | ✓ |
| IL-8 | Interleukin-8 | P10145 | *IL8* | 4 | 74606223 | 74609433 | 1 | ✓ | ✓ | ✓ |
| IL16 | Pro-interleukin-16 | Q14005 | *IL16* | 15 | 81489220 | 81605104 | 1 | ✓ | ✓ | ✓ |
| ITGB1BP2 | Melusin | Q9UKP3 | *ITGB1BP2* | X | 70521584 | 70525221 | 1 | ✓ | | |
| KIM-1 | Kidney injury molecule 1 | Q96D42 | *HAVCR1* | 5 | 156456437 | 156485966 | -1 | ✓ | ✓ | ✓ |
| KLK6 | Kallikrein-6 | Q92876 | *KLK6* | 19 | 51461887 | 51472929 | -1 | ✓ | ✓ | ✓ |
| LEP | Leptin | P41159 | *LEP* | 7 | 127881337 | 127897681 | 1 | ✓ | ✓ | ✓ |
| LOX-1 | Lectin-like oxidized LDL receptor 1 | P78380 | *OLR1* | 12 | 10310902 | 10324737 | -1 | ✓ | ✓ | ✓ |
| MB | Myoglobin | P02144 | *MB* | 22 | 36002811 | 36019401 | -1 | ✓ | ✓ | ✓ |
| MCP-1 | Monocyte chemotactic protein 1 | P13500 | *CCL2* | 17 | 32582304 | 32584222 | 1 | ✓ | ✓ | ✓ |
| MMP-1 | Matrix metalloproteinase-1 | P03956 | *MMP1* | 11 | 102660651 | 102668891 | -1 | ✓ | ✓ | ✓ |
| MMP-10 | Matrix metalloproteinase-10 | P09238 | *MMP10* | 11 | 102641234 | 102651359 | -1 | ✓ | ✓ | ✓ |
| MMP-12 | Matrix metalloproteinase-12 | P39900 | *MMP12* | 11 | 102733467 | 102745765 | -1 | ✓ | ✓ | ✓ |
| MMP-3 | Matrix metalloproteinase-3 | P08254 | *MMP3* | 11 | 102706532 | 102714534 | -1 | ✓ | ✓ | ✓ |
| MMP-7 | Matrix metalloproteinase-7 | P09237 | *MMP7* | 11 | 102391239 | 102401484 | -1 | ✓ | | ✓ |
| MPO | Myeloperoxidase | P05164 | *MPO* | 17 | 56347217 | 56358296 | -1 | ✓ | ✓ | ✓ |
| NEMO | NF-kappa-B essential modulator | Q9Y6K9 | *IKBKG* | X | 153770459 | 153793261 | 1 | ✓ | | |
| NT-pro_BNP | N-terminal prohormone brain natriuretic peptide | NA | *NPPB* | 1 | 11917521 | 11918988 | -1 | ✓ | | ✓ |
| OPG | Osteoprotegerin | O00300 | *TNFRSF11B* | 8 | 119935796 | 119964439 | -1 | ✓ | ✓ | ✓ |
| PAPPA | Pappalysin-1 | Q13219 | *PAPPA* | 9 | 118916083 | 119164601 | 1 | ✓ | ✓ | ✓ |
| PAR-1 | Proteinase-activated receptor 1 | P25116 | *F2R* | 5 | 76011868 | 76031606 | 1 | ✓ | ✓ | ✓ |
| PDGF | Platelet-derived growth factor subunit B | P01127 | *PDGFB* | 22 | 39619364 | 39640756 | -1 | ✓ | ✓ | ✓ |
| PECAM-1 | Platelet endothelial cell adhesion molecule | P16284 | *PECAM1* | 17 | 62399863 | 62491136 | -1 | ✓ | ✓ | ✓ |
| PlGF | Placenta growth factor | P49763 | *PGF* | 14 | 75408537 | 75422487 | -1 | ✓ | ✓ | ✓ |
| PRL | Prolactin | P01236 | *PRL* | 6 | 22287480 | 22297730 | -1 | | ✓ | |
| PSGL-1 | P-selectin glycoprotein ligand 1 | Q14242 | *SELPLG* | 12 | 109015686 | 109027735 | -1 | ✓ | ✓ | ✓ |

| | | | | | | | | | | |
|---|---|---|---|---|---|---|---|---|---|---|
| PTX3 | Pentraxin-related protein PTX3 | P26022 | *PTX3* | 3 | 157154578 | 157161417 | 1 | ✓ | | ✓ |
| RAGE | Receptor for advanced glycosylation end products | Q15109 | *AGER* | 6 | 32097222 | 32228531 | -1 | ✓ | ✓ | ✓ |
| REN | Renin | P00797 | *REN* | 1 | 204123944 | 204135465 | -1 | ✓ | ✓ | ✓ |
| RETN | Resistin | Q9HD89 | *RETN* | 19 | 7733930 | 7735334 | 1 | ✓ | ✓ | ✓ |
| SCF | Stem cell factor | P21583 | *KITLG* | 12 | 88890359 | 88974628 | -1 | ✓ | ✓ | ✓ |
| SELE | E-selectin | P16581 | *SELE* | 1 | 169691782 | 169703203 | -1 | ✓ | ✓ | ✓ |
| SIRT2 | SIR2-like protein 2 | Q8IXJ6 | *SIRT2* | 19 | 39369197 | 39390502 | -1 | ✓ | | ✓ |
| SPON1 | Spondin-1 | Q9HCB6 | *SPON1* | 11 | 13984236 | 14289351 | 1 | ✓ | ✓ | ✓ |
| SRC | Proto-oncogene tyrosine-protein kinase Src | P12931 | *SRC* | 20 | 35973088 | 36034453 | 1 | ✓ | ✓ | |
| ST2 | ST2 protein | Q01638 | *IL1RL1* | 2 | 102927962 | 102968497 | 1 | ✓ | ✓ | ✓ |
| t-PA | Tissue-type plasminogen activator | P00750 | *PLAT* | 8 | 42032751 | 42065242 | -1 | ✓ | ✓ | ✓ |
| TF | Tissue factor | P13726 | *F3* | 1 | 94994781 | 95007356 | -1 | ✓ | ✓ | ✓ |
| TIE2 | Angiopoietin-1 receptor | Q02763 | *TEK* | 9 | 27109139 | 27230173 | 1 | ✓ | ✓ | ✓ |
| TM | Thrombomodulin | P07204 | *THBD* | 20 | 23026270 | 23030378 | -1 | ✓ | ✓ | ✓ |
| TNF-R1 | Tumor necrosis factor receptor 1 | P19438 | *TNFRSF1A* | 12 | 6437923 | 6451280 | -1 | ✓ | ✓ | ✓ |
| TNF-R2 | Tumor necrosis factor receptor 2 | P20333 | *TNFRSF1B* | 1 | 12227060 | 12269285 | 1 | ✓ | ✓ | ✓ |
| TNFSF14 | Tumor necrosis factor ligand superfamily member 14 | O43557 | *TNFSF14* | 19 | 6663148 | 6670599 | -1 | ✓ | ✓ | ✓ |
| TRAIL | TNF-related apoptosis-inducing ligand | P50591 | *TNFSF10* | 3 | 172223298 | 172241297 | -1 | ✓ | ✓ | ✓ |
| TRAIL-R2 | TNF-related apoptosis-inducing ligand receptor 2 | O14763 | *TNFRSF10B* | 8 | 22877646 | 22926692 | -1 | ✓ | ✓ | ✓ |
| TRANCE | TNF-related activation-induced cytokine | O14788 | *TNFSF11* | 13 | 43136872 | 43182149 | 1 | ✓ | ✓ | ✓ |
| U-PAR | Urokinase plasminogen activator surface receptor | Q03405 | *PLAUR* | 19 | 44150247 | 44174699 | -1 | ✓ | ✓ | ✓ |
| VEGF-A | Vascular endothelial growth factor A | P15692 | *VEGFA* | 6 | 43738519 | 43754224 | 1 | ✓ | ✓ | ✓ |
| VEGF-D | Vascular endothelial growth factor D | O43915 | *FIGF* | X | 15363713 | 15402498 | -1 | ✓ | ✓ | |

**Supplemental Table 3.** Studies included in the GWAS meta-analysis of circulating protein level from SCALLOP Consortium

The following table is adapted from Folkersen *et al*. 2020[15]

| Study | Reference(s) | Design | Max sample size | GWAS software | GWAS software method | Adjustment |
|---|---|---|---|---|---|---|
| IMPROVE | PMID: 23152477 | Patients with metabolic syndrome | 3403 | Plink 1.9 | dosage | site, age, sex, olink_batch |
| STANLEY | | Bipolar cases and controls | 681 | Plink 1.9 | dosage | age, sex, MDS components, olink batch |
| EpiHealth | PMID: 23435790 | Population-based | 2335 | SNPTEST v.2.5.2 | -frequentist 1 -method 'em' | Age, sex, OLINK plate, MDS component 1-5 |
| PIVUS | PMID: 16141402 | Population-based | 933 | SNPTEST v.2.5.2 | -frequentist 1 -method 'em' | Age, sex, OLINK plate, storage time, MDS component 1-2 |
| ULSAM | PMID: 16030278 | Population-based | 730 | SNPTEST v.2.5.2 | -frequentist 1 -method 'em' | Age, OLINK plate, storage time, MDS component 1-2 |
| INTERVAL | PMID: 27863252 | RCT (blood donors) | 4,987 (post QC) | SNPTEST v2.5.2 | -method expected | Protein abundance was regressed on age, sex, season, plate, bleed to processing time (days), before IVNT. PLINK MDS 1-3 used as covariates in SNPTEST |
| LifeLines-DEEP | PMID: 26319774 | Population-based | 1178 | https://github.com/molgenis/systemsgenetics/tree/master/eqtl-mapping-pipeline | Spearman's correlation | age, gender, smoking status, oral contraceptive usage, blood cell counts |
| NSPHS | PMID:19060911 & 20568910 | Population-based | 965 | ProbABEL/palinear (v1.8.0) | dosage | selection of 159 covariates per protein, also including storage time. |
| STABILITY | PMID: 24678955 | RCT | 2,967 | SNPTEST v2.5.2 | -frequentist 1 -method score | AGE, SEX, PC1, PC2, PC3, PC4 |
| Estonian BB | PMID:24518929 | Population-based | 496 (post QC) | Plink 1.9 | dosage | age, sex, OLINK plate, MDS components 1-10 |
| ORCADES | PMID: 18760389 | Population-based isolate | 971 | GenABEL+RegScan | LMM (Grammar Gamma) + genome scan | age+sex+array+time_in_storage_days+season_of_vene+plate_no+plate_row+plate_column+pc{1:10} |
| VIS | | Population-based isolate | | GenABEL+RegScan | LMM (Grammar Gamma) + genome scan | Age+sex |
| MPP-RES | PMID: 20211303 | Population-based, oversampling of subjects with diabetes and impaired fasting glucose | 882 | SNPTEST v.2.5.2 | -frequentist 1 -method expected | Age, sex, Olink plate |

**Supplemental Table 4.** Studies included in the GWAS meta-analysis of heart failure from HERMES Consortium

The following table is adapted from Shah, et al. (2020)[19]

| Study | Study Design | HF cases | Heart Failure Definition | Physician adjudication | ICD codes | Imaging | Natriuretic peptides | HF treatment |
|---|---|---|---|---|---|---|---|---|
| ARIC | Population-based prospective cohort study | Incident + prevalent | Incident HF was defined as the first HF hospitalization or presence of HF code on death certificate since baseline visit through 2013. Discharge records and death certificates that showed a HF code in any position with International Classification of Diseases Code, Ninth Revision (ICD-9) code 428.x, and deaths with ICD-9/10 codes of either 428.x or I50 were considered as HF. Prevalent Heart Failure was ascertained at first visit. If the participant reported to have taken any medication for heart failure, or qualifies for the Gothenburg Criteria then the participant had prevalent heart failure at baseline. | ✓ | ✓ | | | ✓ |
| BIOSTAT-CHF | Heart failure prospective cohort study | Prevalent | Heart failure was defined based on physician diagnosis, previous documented admission with heart failure requiring diuretic treatment, treatment with furosemide ≥20 mg/day or equivalent. | ✓ | | | | ✓ |
| CHS | Population-based prospective cohort study | Incident | Incident HF events were identified by self-report or administrative data validated by physician's review of medical records, as described in previous reports (PMID: 1669507). In brief, heart failure was defined on the basis of cardiomegaly and pulmonary edema on chest X-ray; or dilated ventricle and wail-motion abnormalities by echocardiography or contrast ventriculography; or congestive failure diagnosed by physician, plus receiving medical treatment (diuretic plus either digitalis, vasodilator or angiotensin converting enzyme inhibitor). | ✓ | ✓ | ✓ | | ✓ |
| COGEN | Healthcare-based prospective cohort study | Incident + prevalent | Inclusion criteria: patients ≥18 years, LVEF <40% or symptoms of clinical HF assessed by a physician including NYHA>1<br>Exclusion criteria: patients with cardiac valvular pathology (e.g. aortic stenosis), HTx, PAH or other structural heart disease | ✓ | | ✓ | | |
| deCODE | Population-based prospective cohort study | Incident + prevalent | Heart failure case status was assigned based on ICD-9 or ICD-10 codes for discharge diagnoses (ICD-10: I50 and subcodes, ICD-9: 428 and subcodes). | | ✓ | | | |
| EGCUT | Population-based prospective cohort study | Incident + prevalent | Heart failure status was assigned based on ICD-10: I50 and subcodes. | | ✓ | | | |
| EPHESUS | Heart failure randomised controlled trial | Prevalent | Patients were recruited 3 to 14 days after acute myocardial infarction according to the following critera: acute myocardial infarction as documented according to standard criteria; left ventricular dysfunction as documented by a left ventricular ejection fraction of 40 percent or lower on echocardiography, radionuclide angiography, or angiography of the left ventricle after the index acute myocardial infarction and before randomization; and heart failure as documented by the presence of pulmonary rales, chest radiography showing pulmonary | ✓ | | ✓ | | |

| | | | | | | | | |
|---|---|---|---|---|---|---|---|---|
| | | | venous congestion, or the presence of a third heart sound. In patients with diabetes who met the criteria for left ventricular dysfunction after acute myocardial infarction, symptoms of heart failure did not have to be demonstrated, since such patients have an increased risk of cardiovascular events similar to that of nondiabetic patients with symptoms of heart failure. | | | | | |
| EPIC-Norfolk | Population-based prospective cohort study | Incident + prevalent | Heart failure definition was based on hospital admission or death record listing Heart failure code - ICD-10: I50 and subcodes | ✓ | ✓ | | | |
| FHS | Population-based prospective cohort study | Incident | Criteria for defining heart failure in the FHS have been described previously (PMID: 5122894, 16837677). In brief, heart failure was considered to be present if two major or one major plus two minor criteria were present in the absence of an alternative explanation for the symptoms and signs. Major criteria are defined as paroxysmal nocturnal dyspnea, orthopnea, jugular venous distention, hepatojugular reflux, pulmonary rales, radiographic evidence of cardiomegaly, acute pulmonary edema, third heart sound, central venous pressure >16 cm of water, and weight loss >4.5 kg during first 5 days of treatment for suspected heart failure. Minor criteria are defined as bilateral ankle edema, nocturnal cough, dyspnea on ordinary exertion, hepatomegaly, pleural effusion, and heart rate >120 beats per minute. | ✓ | | ✓ | | ✓ |
| FINRISK | Population-based prospective cohort study | Incident + prevalent | Individuals with a diagnosis corresponding to heart failure in the nationwide hospital discharge or cause of death registers (ICD-10: I50, I110, I130 and I132; ICD-9: 4029B, 404, 4148, 428; ICD-7: 42700, 42710, 428) or special drug reimbursement for heart failure medications (requires a medical certificate that meets predefined criteria for heart failure). | | ✓ | | | ✓ |
| GoDARTS | Population-based prospective cohort study | Incident + prevalent | HF cases were defined by the presence of at least one of the following criteria:<br>1. Echocardiographic evidence of left ventricular systolic impairment and diuretic prescription<br>2. Admission to hospital with HF and receipt of a loop diuretic prescription<br>Patients who were never prescribed a loop diuretic were not classified as HF cases. Date of HF diagnosis was taken as either the date of the earliest echocardiogram or the date of the earliest admission to hospital for HF. | | ✓ | ✓ | | ✓ |
| GRADE | Prospective cohort study | Prevalent | Inclusion criteria were: patients who were ≥18 years of age with a diagnosis of at least moderate systolic left ventricular dysfunction (EF ≤30%), and who had an ICD at the University of Pittsburgh Medical Center, Emory University Medical Center, Massachusetts General Hospital, Ohio State University Medical Center, Mid-Ohio Cardiology or the Pittsburgh Veterans Affairs Medical Center. Subjects were excluded if they had intractable Class IV heart failure, and conditions (other than HF) that were expected to limit survival to less than 6 months. | ✓ | | ✓ | | |
| LURIC | Population-based prospective cohort study | Incident | A clinical diagnosis of (left) heart failure was defined by the combined presence of symptoms of dyspnea on exertion and cardiac disease with impaired left ventricular function. Left ventricular dysfunction or impaired left ventricular function was defined by imaging techniques, such as echocardiography and left ventricular angiography, and graded semi-quantitatively into normal, minimal, moderate and severe impairment of left ventricular pump function. Based on the underlying cardiac disease, heart failure/left ventricular dysfunction was either of ischaemic (in case of CAD) or of non-ischaemic origin (dilated cardiomyopathy being the most frequent non-CAD disease). Mis- or underreporting of heart failure and/or left ventricular dysfunction was avoided in LURIC since virtually all LURIC participants, except for family members without coronary angiography, underwent echocardiography and left ventricular angiography. | ✓ | | ✓ | | |

| | | | | | | | | |
|---|---|---|---|---|---|---|---|---|
| MDCS | Population-based prospective cohort study | Incident + prevalent | Heart failure was ascertained from the Swedish Hospital Discharge Register using diagnosis codes 427.00, 427.10, and 428.99 for International Classification of Diseases-8th Revision (ICD-8), 428 for the 9th Revision (ICD-9), and I50 and I11.0 for the 10th Revision (ICD-10) as primary diagnosis, according to a previous validation study (PMID:15916919). | | | | | |
| PHFS | Heart failure prospective cohort study | Prevalent | Prevalent heart failure diagnosed by a heart failure cardiologist based on clinical evaluation and cardiac imaging | ✓ | | ✓ | | |
| PIVUS | Population-based prospective cohort study | Incident + prevalent | The medical records for all individuals with heart failure diagnosis in any position in the Swedish hospital discharge register were reviewed by two physicians who were blinded to the baseline data. They classified the cases as definite, questionable, or miscoded according to the European Society of Cardiology recommendations. They considered ICD heart failure codes 427.00, 427.10, 428 (ICD-9), I50 (ICD-10) and hypertensive heart disease with heart failure, I11.0 (ICD-10) as possible diagnosis of heart failure. For further details see PMID:15916919. | ✓ | ✓ | | | |
| PREVEND | Population-based prospective cohort study | Incident + prevalent | Heart failure cases were ascertained using criteria in accordance with the Heart Failure Guidelines of the European Society of Cardiology (ESC). In- and outpatient files were inspected for the presence of heart failure at baseline and for new onset heart failure, by recording signs, symptoms, and objective evidence of heart failure. In total, 586 individual cases were identified as suspected heart failure. An endpoint adjudication committee of seven independent experts evaluated all suspected cases of new onset heart failure. Each case was validated by two different experts by reviewing anonymized clinical charts, hospitalization, and physician office records in order to ascertain the incidence of heart failure. In case of consensus, patients were classified as 'definite new onset heart failure', 'definite no new onset heart failure', or 'definite heart failure, with date of onset before time of recruitment. In case of difference of opinion about an individual case, the committee made a joint decision. | ✓ | | ✓ | | |
| PROSPER | Randomised-controlled trial | Incident + prevalent | Cases were defined by hospitalization for heart failure with a definition based on a combination of symptoms (e.g. shortness of breath) and signs, including chest radiograph with fluid congestion or echocardiogram with severely diminished LV function. All outcomes were adjudicated by an expert committee blinded to randomized study medication and using pre-defined criteria. | ✓ | | ✓ | | |
| Regeneron/Geisinger | Population-based prospective cohort study | Incident + prevalent | Heart failure status was assigned based on ICD-10: I50 and subcodes. | | ✓ | | | |
| Rotterdam study 1 | Population-based prospective cohort study | Incident | Prevalent heart failure at baseline was assessed using a validated score based on the European Society of Cardiology recommendation, identified from hospital discharge diagnoses, and restropective medical records screening. Cases of incident heart failure were obtained by continuously monitoring participants for the occurrence of heart failure during follow-up through general practitioners records and hospital discharge diagnoses. The date of incident heart failure was defined as the day of the first occurrence of symptoms suggestive of heart failure, obtained from the medical records, or the day of receipt of a first prescription for a loop diuretic or an ACEinhibitor indicated for treatment of heart failure, whichever came first. The diagnosis of heart failure was classified as definite, probable, possible, or unlikely in | ✓ | | ✓ | | ✓ |

| | | | | | | | | |
|---|---|---|---|---|---|---|---|---|
| | | | accordance with the criteria from the European Society of Cardiology. Potential cases were ascertained by two research physicians and verified by a cardiologist. Only definite and probable cases were considered in the analyses. | | | | | |
| SHIP | Population-based prospective cohort study | Incident + prevalent | For these analyses heart failure was defnied according to a modified Rotterdam definition (PMID: 10213348). Prevalent HF cases in SHIP were defined as having history of HF (either chest pain during exercise, bypass, heart transplant, atrial flutter or fibrillation, LV hypertophy in individuals aged 45 or older, known MI) and HF symptoms (dyspnoe at exercise or swollen legs at evening) that were not related to bronchitis (bronchitis that occured recently or during the last 12 months). | ✓ | | ✓ | | ✓ |
| SOLID | Randomised-controlled trial | Incident + prevalent | Heart failure status at enrolment was identified from medical record with no specific definition. I HF hospitalizations adjudicated during follow up were defined as admission to hospital or attendance at an acute health care facility for administration of intravenous diuretic treatment, escalation of diuretic doses, and/or inotropes. Confirmation of heart failure diagnosis was obtained by chest imaging demonstrating pulmonary congestion or edema, or, in patients without available chest imaging, at least one of the following: Pulmonary edema, (i.e. rales >1/3 up the lung fields thought to be of cardiac causes), pulmonary capillary wedge pressure >18 mmHg or BNP >500 pg/ml (or NT-terminal prohormone BNP >2500 pg/ml). | ✓ | | ✓ | ✓ | |
| TwinGene | Population-based prospective cohort study | Incident + prevalent | Heart failure status was assigned based on ICD-10: I50; ICD-8 and ICD-9 428 | | ✓ | | | |
| UK Biobank | Population-based prospective cohort study | Incident + prevalent | Individuals with self-reported "HF/pulmonary edema" or "cardiomyopathy"; or who carry an International Classification of Diseases (ICD)-10 or ICD-9 billing code for heart/ventricular failure or cardiomyopathy (ICD-10: I11.0, I13.0, I13.2, I25.5, I42.0, I42.5, I42.8, I42.9, I50.0, I50.1, I50.9; ICD-9: 4254, 4280, 4281, 4289). Individuals with self-reported or an ICD-10 based classification of hypertrophic cardiomyopathy were excluded. | | ✓ | | | |
| ULSAM | Population-based prospective cohort study | Incident + prevalent | The medical records for all individuals with heart failure diagnosis in any position in the Swedish hospital discharge register were reviewed by two physicians who were blinded to the baseline data. They classified the cases as definite, questionable, or miscoded according to the European Society of Cardiology recommendations. They considered ICD heart failure codes 427.00, 427.10, 428 (ICD-9), I50 (ICD-10) and hypertensive heart disease with heart failure, I11.0 (ICD-10) as possible diagnosis of heart failure. For details, please see Ingelsson E, et al Eur J Heart Fail. 2005 Aug;7(5):787-91 | ✓ | ✓ | ✓ | | |
| WGHS | Population-based prospective cohort study | Incident | Heart failure cases were ascertained by cardiologists from medical records. Cases of incident nonfatal HF were confirmed if either the Framingham Heart Study (mainly physical examination and radiographic data) or Cardiovascular Health Study criteria (predominantly based on the treating physician's diagnosis and use of specific therapy) were met. Fatal HF cases included those not identified as a case of HF prior to death and classified into “Definite” or “Probable” fatal HF based on medical records and death certificate with next-of-kin or physician confirmation. | ✓ | | ✓ | | ✓ |

**Supplemental Table 5.** Summary of GWAS summary statistics used in the primary and cross-trait MR analyses

| Trait | Description | Cohort / Consortium | Study | URL |
|---|---|---|---|---|
| Plasma proteins | a GWAS meta-analysis of 90 cardiovascular-related proteins measured using the Olink Proximity Extension Assay CVD-I panel in 30,931 subjects across 14 studies | SCALLOP | Folkersen et al (2020)[15] | https://doi.org/10.1038/s42255-020-00287-2 |
| Heart failure | a GWAS meta-analysis of 47,309 heart failure cases and 930,014 controls from 26 cohorts of European ancestry | HERMES | Shah et al (2020)[19] | https://doi.org/10.1038/s41467-019-13690-5 |
| Coronary artery disease | a multi-ancestry GWAS meta-analysis of 60,801 CAD cases and 123,504 controls from 48 studies | CARDIoGRAMplusC4D | Nikpay et al (2015)[40] | https://doi.org/10.1038/ng.3396 |
| Atrial fibrillation | a multi-ancestry GWAS meta-analysis of 65,446 AF cases and more than 522,000 controls from more than 50 studies | AFGen | Roselli et al (2018)[41] | https://doi.org/10.1038/s41588-018-0133-9 |
| eGFR | a multi-ancestry GWAS meta-analysis of estimated glomerular filtration rate from 1,046,070 participants | CKDGen | Wuttke M et al (2019)[42] | https://doi.org/10.1038/s41588-019-0407-x |
| Systolic and diastolic blood pressure | a GWAS meta-analysis of over 1 million people of European ancestry to investigate high blood pressure traits | UK Biobank + ICBP | Evangelou et al (2018)[43] | https://dx.doi.org/10.1038%2Fs41588-018-0205-x |
| Type 2 diabetes | a GWAS meta-analysis of 74,124 type 2 diabetes cases and 824,006 controls of European ancestry. | DIAGRAM | Mahajan A et al (2018)[44] | https://doi.org/10.1038/s41588-018-0241-6 |
| Body mass index | a multi-ancestry GWAS meta-analysis of body mass index from 694,649 participants of European ancestry | GIANT + UK Biobank | Pulit S et al (2019)[45] | https://doi.org/10.1093/hmg/ddy327 |

**Supplemental Table 6.** Study-level and meta-analysis estimates of observational associations between circulating protein levels and incident HF

| Protein | Risk ratio of incident HF [95% confidence interval] | | | | | *P* meta-analysis | *P* heterogeneity |
|---|---|---|---|---|---|---|---|
| | HOMAGE Discovery | HOMAGE Replication | PIVUS | ULSAM | Meta-analysis | | |
| ADM | 1.69 [1.40-2.05] | 1.39 [1.17-1.65] | 1.52 [1.17-1.97] | 1.59 [1.27-1.99] | 1.53 [1.39-1.70] | 1.49E-16 | 4.98E-01 |
| AGRP | 1.41 [1.20-1.65] | 1.45 [1.17-1.79] | 1.31 [1.03-1.65] | 1.43 [1.16-1.76] | 1.41 [1.27-1.55] | 1.85E-11 | 9.31E-01 |
| Beta-NGF | 1.25 [1.07-1.46] | 1.06 [0.89-1.26] | | | 1.16 [1.03-1.31] | 1.12E-02 | 1.67E-01 |
| BNP | 1.85 [1.56-2.19] | 2.01 [1.67-2.42] | | | 1.92 [1.70-2.18] | 1.91E-24 | 5.19E-01 |
| CA-125 | | | 1.09 [0.87-1.36] | 1.08 [0.87-1.34] | 1.08 [0.93-1.27] | 3.08E-01 | 9.54E-01 |
| CASP-8 | 1.39 [1.13-1.72] | 1.28 [1.05-1.56] | 1.24 [0.97-1.59] | 0.81 [0.65-1.01] | 1.17 [1.05-1.30] | 4.81E-03 | 2.97E-03 |
| CCL20 | 1.26 [1.08-1.46] | 1.08 [0.92-1.26] | 1.22 [1.00-1.49] | 1.36 [1.14-1.62] | 1.22 [1.12-1.33] | 3.86E-06 | 2.76E-01 |
| CCL3 | 1.12 [0.95-1.31] | 1.15 [0.90-1.47] | 1.29 [1.07-1.56] | 1.09 [0.89-1.35] | 1.16 [1.05-1.28] | 2.64E-03 | 6.21E-01 |
| CCL4 | 1.13 [0.97-1.31] | 0.98 [0.84-1.15] | 1.17 [0.96-1.43] | 0.94 [0.76-1.16] | 1.05 [0.97-1.15] | 2.42E-01 | 2.87E-01 |
| CD40 | 1.61 [1.35-1.92] | 1.15 [0.96-1.37] | 1.47 [1.20-1.81] | 1.22 [0.99-1.50] | 1.36 [1.23-1.49] | 4.34E-10 | 3.83E-02 |
| CD40-L | 1.15 [0.98-1.35] | 1.11 [0.94-1.30] | 1.07 [0.85-1.36] | 0.88 [0.73-1.05] | 1.05 [0.96-1.15] | 2.72E-01 | 1.30E-01 |
| CHI3L1 | 1.32 [1.13-1.53] | 1.19 [1.01-1.40] | 1.33 [1.08-1.63] | 1.25 [1.02-1.54] | 1.27 [1.16-1.39] | 1.57E-07 | 7.87E-01 |
| CSF-1 | 1.36 [1.14-1.62] | 1.13 [0.91-1.40] | 1.54 [1.22-1.95] | 1.33 [1.06-1.66] | 1.33 [1.20-1.47] | 9.51E-08 | 2.91E-01 |
| CSTB | 1.76 [1.47-2.10] | 1.22 [1.05-1.42] | | | 1.42 [1.26-1.59] | 2.94E-09 | 2.18E-03 |
| CTSD | 1.15 [0.98-1.36] | 1.03 [0.88-1.20] | 1.17 [0.94-1.46] | 1.22 [0.98-1.51] | 1.12 [1.03-1.23] | 1.24E-02 | 5.89E-01 |
| CTSL1 | 1.22 [1.02-1.46] | 1.14 [0.98-1.34] | 1.35 [1.08-1.69] | 1.20 [0.97-1.48] | 1.21 [1.10-1.32] | 7.13E-05 | 6.74E-01 |
| CX3CL1 | 1.30 [1.07-1.57] | 1.12 [0.91-1.36] | 1.32 [1.06-1.66] | 1.10 [0.89-1.36] | 1.21 [1.09-1.34] | 3.80E-04 | 4.94E-01 |
| CXCL1 | 1.05 [0.89-1.24] | 1.06 [0.87-1.29] | 0.86 [0.69-1.06] | 1.25 [1.01-1.54] | 1.04 [0.95-1.15] | 3.79E-01 | 1.01E-01 |
| CXCL16 | 1.38 [1.16-1.65] | 1.13 [0.96-1.33] | 1.27 [1.01-1.60] | 1.10 [0.89-1.36] | 1.21 [1.11-1.33] | 4.65E-05 | 2.74E-01 |
| CXCL6 | 1.17 [0.99-1.39] | 0.99 [0.83-1.17] | 0.95 [0.76-1.19] | 0.96 [0.77-1.20] | 1.04 [0.94-1.14] | 4.88E-01 | 3.58E-01 |
| Dkk-1 | 1.22 [1.01-1.48] | 1.15 [0.98-1.37] | 0.82 [0.66-1.03] | 0.97 [0.78-1.20] | 1.06 [0.96-1.16] | 2.36E-01 | 3.35E-02 |
| ECP | | | 1.35 [1.08-1.68] | 0.90 [0.74-1.10] | 1.07 [0.93-1.24] | 3.33E-01 | 7.04E-03 |
| EGF | | | 0.81 [0.64-1.02] | 0.84 [0.68-1.03] | 0.83 [0.71-0.96] | 1.44E-02 | 8.17E-01 |
| EN-RAGE | 1.27 [1.08-1.50] | 1.57 [1.26-1.96] | 1.18 [0.96-1.46] | 1.08 [0.87-1.34] | 1.26 [1.14-1.38] | 4.04E-06 | 9.61E-02 |
| ESM-1 | | | 1.11 [0.88-1.39] | 1.23 [0.99-1.52] | 1.17 [1.00-1.38] | 5.18E-02 | 5.30E-01 |
| FABP4 | 1.80 [1.50-2.16] | 1.56 [1.28-1.89] | 1.72 [1.32-2.24] | 1.33 [1.09-1.63] | 1.59 [1.43-1.76] | 9.95E-19 | 1.59E-01 |
| FAS | 1.36 [1.14-1.62] | 1.25 [1.05-1.49] | 1.15 [0.94-1.40] | 1.34 [1.12-1.61] | 1.28 [1.17-1.40] | 1.13E-07 | 6.08E-01 |
| FGF-23 | 1.54 [1.31-1.81] | 1.65 [1.34-2.02] | 1.30 [1.08-1.56] | 1.56 [1.31-1.86] | 1.50 [1.37-1.64] | 6.63E-19 | 3.28E-01 |
| FS | 1.17 [0.96-1.43] | 1.23 [1.01-1.50] | 1.35 [1.08-1.68] | 1.39 [1.13-1.72] | 1.28 [1.15-1.41] | 3.61E-06 | 6.23E-01 |
| GAL | | | 0.96 [0.76-1.21] | 1.30 [1.04-1.61] | 1.12 [0.96-1.31] | 1.61E-01 | 5.85E-02 |
| Gal-3 | 1.35 [1.11-1.63] | 1.11 [0.92-1.35] | 1.21 [0.95-1.56] | 1.19 [0.95-1.49] | 1.21 [1.09-1.35] | 3.53E-04 | 5.65E-01 |
| GDF-15 | 1.90 [1.59-2.26] | 1.49 [1.26-1.77] | 1.72 [1.39-2.13] | 1.61 [1.33-1.96] | 1.66 [1.52-1.83] | 4.00E-27 | 2.61E-01 |
| GH | 0.95 [0.82-1.10] | 1.09 [0.92-1.29] | 1.05 [0.83-1.32] | 1.22 [0.99-1.51] | 1.05 [0.96-1.15] | 2.95E-01 | 2.74E-01 |
| HB-EGF | 1.15 [0.95-1.40] | 1.17 [1.00-1.37] | 1.15 [0.92-1.43] | 0.98 [0.79-1.22] | 1.13 [1.02-1.25] | 1.44E-02 | 7.11E-01 |
| HGF | 1.65 [1.37-1.98] | 1.25 [1.06-1.47] | 1.43 [1.17-1.75] | 1.14 [0.92-1.41] | 1.36 [1.24-1.49] | 1.64E-10 | 4.57E-02 |
| hK11 | | | 1.28 [1.08-1.51] | 1.17 [0.95-1.44] | 1.23 [1.08-1.41] | 1.53E-03 | 5.05E-01 |
| HSP | 1.63 [1.25-2.12] | 1.20 [1.01-1.42] | | | 1.31 [1.14-1.52] | 2.13E-04 | 5.79E-02 |
| IL-18 | 1.19 [1.01-1.39] | 1.24 [1.06-1.45] | 1.26 [1.00-1.58] | 1.30 [1.05-1.61] | 1.24 [1.13-1.36] | 4.33E-06 | 9.28E-01 |
| IL-1ra | 1.50 [1.25-1.80] | 1.24 [1.07-1.43] | 1.21 [0.98-1.49] | 0.94 [0.75-1.18] | 1.24 [1.13-1.36] | 4.86E-06 | 2.08E-02 |
| IL-27 | 1.27 [1.02-1.57] | 1.46 [1.13-1.88] | 1.28 [1.01-1.61] | 1.34 [1.08-1.65] | 1.33 [1.18-1.49] | 1.15E-06 | 8.52E-01 |
| IL-6 | 1.51 [1.29-1.76] | 1.21 [1.03-1.43] | 1.53 [1.29-1.81] | 1.18 [0.97-1.43] | 1.36 [1.25-1.48] | 9.45E-13 | 5.31E-02 |
| IL-6RA | 1.23 [1.03-1.46] | 1.10 [0.94-1.29] | 1.05 [0.84-1.32] | 1.04 [0.84-1.28] | 1.12 [1.01-1.23] | 2.36E-02 | 6.15E-01 |
| IL-8 | 1.18 [1.02-1.37] | 1.12 [0.94-1.33] | 1.25 [1.03-1.50] | 1.04 [0.84-1.30] | 1.15 [1.06-1.26] | 1.48E-03 | 6.05E-01 |
| IL16 | 1.36 [1.14-1.62] | 1.31 [1.11-1.55] | 1.28 [1.02-1.61] | 1.04 [0.83-1.29] | 1.27 [1.15-1.40] | 1.57E-06 | 3.40E-01 |
| ITGB1BP2 | 1.23 [1.03-1.48] | 1.06 [0.86-1.30] | | | 1.16 [1.01-1.32] | 3.60E-02 | 2.88E-01 |

| | | | | | | | |
|---|---|---|---|---|---|---|---|
| KIM-1 | 1.37 [1.17-1.60] | 1.46 [1.21-1.75] | 1.62 [1.30-2.01] | 1.56 [1.30-1.87] | 1.48 [1.35-1.62] | 5.14E-17 | 5.91E-01 |
| KLK6 | 1.04 [0.85-1.27] | 0.93 [0.79-1.09] | 1.08 [0.86-1.35] | 1.20 [0.97-1.49] | 1.04 [0.94-1.14] | 4.89E-01 | 3.07E-01 |
| LEP | 1.23 [1.03-1.47] | 1.29 [1.05-1.58] | 1.35 [1.01-1.80] | 1.25 [1.00-1.56] | 1.27 [1.14-1.41] | 1.50E-05 | 9.54E-01 |
| LOX-1 | 1.30 [1.08-1.57] | 1.37 [1.15-1.64] | 1.26 [1.04-1.52] | 0.99 [0.80-1.22] | 1.23 [1.12-1.35] | 1.32E-05 | 8.72E-02 |
| MB | 1.15 [0.98-1.37] | 0.98 [0.83-1.16] | 1.16 [0.92-1.46] | 1.40 [1.14-1.73] | 1.14 [1.04-1.25] | 5.87E-03 | 7.42E-02 |
| MCP-1 | 1.21 [1.04-1.40] | 1.10 [0.93-1.32] | 1.28 [1.05-1.56] | 0.97 [0.78-1.21] | 1.15 [1.05-1.26] | 1.96E-03 | 2.79E-01 |
| MMP-1 | 1.19 [1.01-1.39] | 1.13 [0.95-1.35] | 1.13 [0.91-1.42] | 1.02 [0.81-1.27] | 1.13 [1.03-1.25] | 1.12E-02 | 8.04E-01 |
| MMP-10 | 1.23 [1.06-1.43] | 0.92 [0.77-1.10] | 1.42 [1.15-1.74] | 1.13 [0.93-1.38] | 1.15 [1.05-1.26] | 1.76E-03 | 1.31E-02 |
| MMP-12 | 1.45 [1.23-1.69] | 1.38 [1.17-1.63] | 1.56 [1.27-1.92] | 1.47 [1.20-1.81] | 1.45 [1.33-1.59] | 6.16E-16 | 8.37E-01 |
| MMP-3 | 1.33 [1.11-1.59] | 1.14 [0.95-1.37] | 1.22 [0.95-1.56] | 1.21 [0.99-1.47] | 1.22 [1.11-1.35] | 6.23E-05 | 7.04E-01 |
| MMP-7 | 1.47 [1.03-2.12] | 1.23 [0.92-1.66] | | | 1.32 [1.05-1.65] | 1.53E-02 | 4.47E-01 |
| MPO | 1.24 [1.06-1.46] | 1.10 [0.94-1.28] | 1.04 [0.83-1.30] | 0.93 [0.75-1.16] | 1.11 [1.01-1.21] | 3.24E-02 | 2.06E-01 |
| NEMO | 1.29 [1.09-1.53] | 1.10 [0.90-1.34] | | | 1.21 [1.06-1.37] | 4.14E-03 | 2.33E-01 |
| NT-pro_BNP | 1.74 [1.47-2.07] | 2.01 [1.65-2.46] | | | 1.85 [1.63-2.10] | 5.54E-21 | 2.76E-01 |
| OPG | 1.50 [1.27-1.78] | 1.20 [1.01-1.42] | 1.50 [1.21-1.87] | 1.31 [1.06-1.62] | 1.37 [1.24-1.50] | 7.33E-11 | 2.36E-01 |
| PAPPA | 1.15 [0.97-1.37] | 1.11 [0.91-1.34] | 0.87 [0.69-1.10] | 0.96 [0.78-1.20] | 1.04 [0.94-1.15] | 4.11E-01 | 2.15E-01 |
| PAR-1 | 1.46 [1.19-1.79] | 1.40 [1.14-1.72] | 1.44 [1.15-1.82] | 1.29 [1.03-1.60] | 1.40 [1.26-1.56] | 9.35E-10 | 8.63E-01 |
| PDGF | 1.16 [0.98-1.37] | 1.13 [0.92-1.38] | 0.86 [0.69-1.07] | 0.92 [0.75-1.14] | 1.03 [0.94-1.14] | 5.06E-01 | 1.02E-01 |
| PECAM-1 | 1.28 [1.07-1.53] | 1.03 [0.88-1.20] | 1.21 [0.96-1.52] | 0.97 [0.78-1.20] | 1.11 [1.01-1.22] | 3.46E-02 | 1.41E-01 |
| PlGF | 1.50 [1.25-1.79] | 1.41 [1.18-1.69] | 1.60 [1.29-1.99] | 1.37 [1.12-1.68] | 1.46 [1.33-1.61] | 1.46E-14 | 7.32E-01 |
| PRL | | | 1.07 [0.86-1.34] | 1.27 [1.02-1.58] | 1.16 [1.00-1.35] | 5.36E-02 | 2.70E-01 |
| PSGL-1 | 1.05 [0.90-1.22] | 1.21 [0.95-1.53] | 1.06 [0.85-1.33] | 1.17 [0.93-1.45] | 1.10 [1.00-1.22] | 6.07E-02 | 7.19E-01 |
| PTX3 | 1.20 [0.99-1.46] | 1.26 [0.97-1.62] | | | 1.22 [1.05-1.43] | 1.17E-02 | 7.68E-01 |
| RAGE | 1.06 [0.90-1.24] | 1.12 [0.95-1.32] | 1.19 [0.95-1.50] | 1.20 [0.96-1.49] | 1.12 [1.02-1.24] | 1.45E-02 | 7.87E-01 |
| REN | 1.29 [1.10-1.52] | 1.51 [1.27-1.80] | 1.14 [0.91-1.44] | 1.20 [0.96-1.51] | 1.31 [1.19-1.44] | 3.07E-08 | 2.03E-01 |
| RETN | 1.46 [1.25-1.71] | 1.22 [1.03-1.44] | 1.13 [0.90-1.42] | 1.07 [0.86-1.33] | 1.26 [1.14-1.38] | 1.53E-06 | 9.10E-02 |
| SCF | 0.86 [0.71-1.04] | 1.14 [0.97-1.35] | 0.73 [0.59-0.90] | 0.89 [0.73-1.09] | 0.92 [0.84-1.02] | 1.01E-01 | 7.85E-03 |
| SELE | 1.20 [1.02-1.40] | 1.17 [0.99-1.39] | 1.20 [0.95-1.51] | 0.90 [0.72-1.12] | 1.13 [1.03-1.24] | 1.16E-02 | 1.71E-01 |
| SIRT2 | 1.24 [1.05-1.48] | 1.09 [0.90-1.33] | | | 1.17 [1.03-1.33] | 1.27E-02 | 3.19E-01 |
| SPON1 | 1.52 [1.25-1.84] | 1.34 [1.08-1.66] | 1.54 [1.22-1.95] | 1.48 [1.20-1.82] | 1.47 [1.32-1.63] | 1.50E-12 | 8.07E-01 |
| SRC | 1.22 [1.03-1.45] | 1.08 [0.90-1.30] | 0.94 [0.74-1.19] | 0.87 [0.71-1.08] | 1.05 [0.95-1.15] | 3.41E-01 | 7.34E-02 |
| ST2 | 1.23 [1.05-1.46] | 1.26 [1.06-1.50] | 1.34 [1.07-1.70] | 1.31 [1.07-1.62] | 1.27 [1.16-1.40] | 3.74E-07 | 9.28E-01 |
| t-PA | 1.17 [0.97-1.40] | 0.99 [0.85-1.16] | 1.37 [1.07-1.75] | 1.24 [1.00-1.53] | 1.13 [1.03-1.25] | 9.84E-03 | 1.08E-01 |
| TF | 1.21 [1.01-1.45] | 1.26 [1.02-1.55] | 1.16 [0.94-1.44] | 1.02 [0.82-1.26] | 1.18 [1.06-1.31] | 2.44E-03 | 6.81E-01 |
| TIE2 | 1.04 [0.85-1.27] | 1.20 [0.94-1.51] | 0.92 [0.73-1.16] | 0.99 [0.80-1.23] | 1.04 [0.92-1.17] | 5.64E-01 | 4.83E-01 |
| TM | 1.38 [1.13-1.70] | 1.23 [1.01-1.49] | 1.09 [0.86-1.39] | 1.08 [0.87-1.34] | 1.20 [1.08-1.33] | 6.29E-04 | 3.09E-01 |
| TNF-R1 | 1.68 [1.39-2.02] | 1.33 [1.13-1.57] | 1.64 [1.32-2.03] | 1.32 [1.06-1.64] | 1.47 [1.34-1.62] | 3.88E-15 | 1.54E-01 |
| TNF-R2 | 1.62 [1.35-1.96] | 1.33 [1.12-1.57] | 1.47 [1.18-1.82] | 1.25 [1.01-1.55] | 1.41 [1.28-1.56] | 1.59E-12 | 2.53E-01 |
| TNFSF14 | 1.24 [1.03-1.48] | 1.12 [0.95-1.32] | 1.18 [0.94-1.49] | 0.89 [0.72-1.10] | 1.11 [1.00-1.22] | 4.14E-02 | 1.11E-01 |
| TRAIL | 1.06 [0.90-1.26] | 0.69 [0.57-0.85] | 1.16 [0.93-1.46] | 1.03 [0.83-1.27] | 0.96 [0.87-1.06] | 4.19E-01 | 1.07E-03 |
| TRAIL-R2 | 1.93 [1.59-2.33] | 1.91 [1.55-2.36] | 1.45 [1.26-1.68] | 1.51 [1.23-1.85] | 1.64 [1.50-1.79] | 1.33E-26 | 4.18E-02 |
| TRANCE | 0.91 [0.78-1.07] | 0.87 [0.72-1.05] | 1.04 [0.83-1.30] | 0.97 [0.79-1.20] | 0.93 [0.84-1.03] | 1.45E-01 | 6.66E-01 |
| U-PAR | 1.75 [1.45-2.10] | 1.33 [1.12-1.58] | 1.81 [1.44-2.27] | 1.37 [1.09-1.72] | 1.53 [1.39-1.70] | 3.72E-17 | 5.67E-02 |
| VEGF-A | 1.67 [1.41-1.98] | 1.24 [1.05-1.48] | 1.26 [1.01-1.57] | 1.04 [0.85-1.28] | 1.31 [1.19-1.44] | 1.15E-08 | 3.52E-03 |
| VEGF-D | 1.30 [1.04-1.61] | 1.44 [1.17-1.78] | 0.99 [0.79-1.23] | 1.40 [1.12-1.75] | 1.23 [1.11-1.36] | 5.95E-05 | 2.14E-02 |

**Supplemental Table 7.** *Cis*- Mendelian randomization (MR) analysis results

LD = linkage disequilibrium; OR = odds ratio

| Protein | Survived multiple testing in observational analysis | Primary *cis*-MR modelling for residual correlation between instruments ($P$ < 1e-4, LD $r^2$ < 0.4) | | | Conventional *cis*-MR with strict instrument selection parameters ($P$ < 5e-8, LD $r^2$ < 0.05) | | | |
|---|---|---|---|---|---|---|---|---|
| | | N instrument | OR [95% CI] | *P* MR estimate | N instrument | OR [95% CI] | *P* MR estimate | *P* instrument heterogeneity |
| ADM | TRUE | 17 | 0.93 [0.89-0.96] | 7.56E-06 | 4 | 0.92 [0.85-0.98] | 1.68E-02 | 5.96E-01 |
| AGRP | TRUE | 3 | 0.96 [0.86-1.07] | 4.46E-01 | 1 | 0.96 [0.80-1.15] | 6.45E-01 | |
| CCL20 | TRUE | 8 | 1.09 [1.01-1.17] | 2.39E-02 | 1 | 1.00 [0.84-1.20] | 1.00E+00 | |
| CD40 | TRUE | 71 | 0.96 [0.94-0.97] | 4.83E-13 | 13 | 0.97 [0.94-1.00] | 7.23E-02 | 1.23E-01 |
| CHI3L1 | TRUE | 81 | 0.97 [0.96-0.98] | 6.91E-07 | 16 | 0.96 [0.94-0.98] | 1.60E-04 | 3.07E-01 |
| CSF-1 | TRUE | 28 | 1.10 [1.08-1.11] | 5.01E-35 | 2 | 1.07 [1.00-1.14] | 3.54E-02 | 9.84E-01 |
| CSTB | TRUE | 93 | 0.99 [0.98-1.00] | 8.79E-02 | 20 | 1.00 [0.98-1.03] | 9.59E-01 | 2.27E-01 |
| CTSL1 | TRUE | 10 | 0.87 [0.83-0.91] | 8.13E-09 | 1 | 0.89 [0.78-1.01] | 8.05E-02 | |
| CX3CL1 | TRUE | 43 | 1.01 [0.99-1.03] | 4.43E-01 | 9 | 1.02 [0.97-1.08] | 4.69E-01 | 6.84E-01 |
| CXCL16 | TRUE | 44 | 1.09 [1.05-1.12] | 2.24E-07 | 5 | 1.00 [0.93-1.07] | 9.22E-01 | 3.17E-01 |
| EN-RAGE | TRUE | 10 | 0.93 [0.89-0.97] | 1.11E-03 | 2 | 1.00 [0.91-1.09] | 9.68E-01 | 5.13E-01 |
| FABP4 | TRUE | 9 | 1.03 [0.97-1.09] | 3.51E-01 | 2 | 0.97 [0.82-1.13] | 6.78E-01 | 5.25E-01 |
| FAS | TRUE | 54 | 0.99 [0.98-1.01] | 4.55E-01 | 8 | 1.00 [0.95-1.05] | 8.74E-01 | 4.89E-01 |
| FGF-23 | TRUE | 5 | 0.80 [0.71-0.89] | 9.94E-05 | 1 | 0.79 [0.62-1.02] | 6.60E-02 | |
| FS | TRUE | 14 | 1.07 [1.00-1.15] | 5.28E-02 | 1 | 0.90 [0.75-1.08] | 2.78E-01 | |
| Gal-3 | TRUE | 47 | 1.03 [1.01-1.05] | 8.48E-04 | 9 | 1.02 [0.98-1.06] | 4.50E-01 | 1.97E-01 |
| GDF-15 | TRUE | 44 | 0.98 [0.97-1.00] | 7.52E-02 | 7 | 0.98 [0.93-1.03] | 3.96E-01 | 2.31E-01 |
| HGF | TRUE | 11 | 1.13 [1.08-1.18] | 2.92E-07 | 2 | 1.09 [0.97-1.22] | 1.45E-01 | 7.07E-01 |
| HSP | TRUE | 38 | 0.96 [0.94-0.98] | 4.90E-04 | 10 | 0.99 [0.95-1.03] | 6.35E-01 | 5.59E-01 |
| IL-18 | TRUE | 21 | 1.05 [1.01-1.08] | 4.23E-03 | 3 | 1.03 [0.96-1.09] | 4.11E-01 | 7.20E-01 |
| IL-1ra | TRUE | 49 | 0.97 [0.94-1.00] | 3.81E-02 | 8 | 1.01 [0.96-1.07] | 6.48E-01 | 2.08E-01 |
| IL16 | TRUE | 33 | 1.00 [0.99-1.01] | 8.27E-01 | 7 | 0.99 [0.96-1.03] | 7.24E-01 | 1.04E-01 |
| KIM-1 | TRUE | 83 | 1.02 [1.01-1.03] | 7.99E-06 | 20 | 1.03 [1.00-1.05] | 2.39E-02 | 1.66E-01 |
| LEP | TRUE | 3 | 1.12 [0.98-1.26] | 8.97E-02 | 1 | 1.05 [0.85-1.29] | 6.72E-01 | |
| LOX-1 | TRUE | 8 | 0.93 [0.84-1.04] | 1.98E-01 | 1 | 0.88 [0.73-1.08] | 2.24E-01 | |
| MMP-12 | TRUE | 114 | 0.98 [0.97-0.99] | 4.13E-04 | 24 | 0.98 [0.96-1.00] | 5.22E-02 | 6.04E-01 |
| MMP-3 | TRUE | 125 | 1.03 [1.02-1.03] | 1.02E-12 | 19 | 1.03 [1.01-1.05] | 4.23E-03 | 9.38E-01 |
| NT-pro_BNP | TRUE | 23 | 1.00 [0.98-1.01] | 6.02E-01 | 1 | 0.99 [0.92-1.07] | 8.51E-01 | |
| OPG | TRUE | 38 | 1.04 [1.02-1.06] | 2.88E-04 | 5 | 1.04 [0.98-1.11] | 2.08E-01 | 6.61E-01 |
| PAR-1 | TRUE | 25 | 1.09 [1.06-1.12] | 8.15E-12 | 4 | 1.05 [0.97-1.13] | 2.46E-01 | 8.14E-01 |
| PIGF | TRUE | 16 | 1.05 [1.01-1.09] | 1.13E-02 | 1 | 1.02 [0.89-1.17] | 7.49E-01 | |
| REN | TRUE | 13 | 0.99 [0.92-1.06] | 7.43E-01 | 3 | 1.04 [0.96-1.12] | 3.06E-01 | 5.49E-01 |
| RETN | TRUE | 27 | 1.00 [0.96-1.03] | 8.58E-01 | 5 | 1.04 [0.96-1.13] | 3.06E-01 | 9.45E-02 |
| SPON1 | TRUE | 46 | 0.95 [0.94-0.96] | 1.50E-15 | 6 | 0.95 [0.88-1.02] | 1.31E-01 | 1.63E-01 |
| ST2 | TRUE | 81 | 1.00 [0.99-1.00] | 4.20E-02 | 25 | 0.99 [0.97-1.01] | 5.16E-01 | 2.15E-01 |
| TNF-R1 | TRUE | 3 | 1.01 [0.89-1.15] | 9.13E-01 | 2 | 1.00 [0.87-1.15] | 9.64E-01 | 9.34E-01 |

| | | | | | | | | |
|---|---|---|---|---|---|---|---|---|
| TNF-R2 | TRUE | 16 | 1.04 [1.00-1.07] | 4.60E-02 | 3 | 1.06 [0.97-1.15] | 2.26E-01 | 4.11E-01 |
| TRAIL-R2 | TRUE | 52 | 0.99 [0.98-1.01] | 3.49E-01 | 8 | 0.99 [0.95-1.03] | 6.35E-01 | 9.78E-02 |
| U-PAR | TRUE | 23 | 0.97 [0.91-1.03] | 2.69E-01 | 4 | 0.96 [0.86-1.08] | 5.23E-01 | 2.64E-01 |
| VEGF-A | TRUE | 34 | 1.01 [1.00-1.02] | 2.02E-01 | 10 | 1.01 [0.97-1.04] | 6.93E-01 | 1.06E-01 |
| Beta-NGF | FALSE | 3 | 1.16 [1.03-1.29] | 1.18E-02 | 0 | | | |
| CA-125 | FALSE | 16 | 1.03 [0.98-1.08] | 2.14E-01 | 2 | 0.99 [0.91-1.08] | 8.18E-01 | 1.23E-02 |
| CASP-8 | FALSE | 10 | 1.01 [0.96-1.07] | 6.06E-01 | 1 | 1.01 [0.90-1.14] | 8.06E-01 | |
| CCL3 | FALSE | 49 | 1.04 [1.02-1.07] | 4.26E-05 | 13 | 1.03 [0.99-1.07] | 1.08E-01 | 1.52E-01 |
| CCL4 | FALSE | 25 | 1.10 [1.04-1.16] | 6.06E-04 | 6 | 1.04 [0.96-1.12] | 3.29E-01 | 1.32E-01 |
| CTSD | FALSE | 90 | 0.97 [0.96-0.99] | 4.02E-04 | 9 | 1.00 [0.97-1.04] | 8.78E-01 | 8.85E-01 |
| CXCL1 | FALSE | 28 | 1.02 [1.01-1.04] | 7.46E-03 | 8 | 1.01 [0.97-1.05] | 6.68E-01 | 7.38E-01 |
| CXCL6 | FALSE | 56 | 1.03 [1.02-1.04] | 1.52E-08 | 13 | 1.04 [1.02-1.06] | 1.91E-04 | 9.28E-01 |
| Dkk-1 | FALSE | 10 | 0.83 [0.73-0.94] | 2.65E-03 | 1 | 0.81 [0.68-0.96] | 1.42E-02 | |
| ECP | FALSE | 11 | 1.03 [1.00-1.06] | 6.78E-02 | 1 | 1.05 [0.98-1.14] | 1.85E-01 | |
| EGF | FALSE | 4 | 0.97 [0.87-1.08] | 5.64E-01 | 1 | 0.97 [0.86-1.09] | 5.97E-01 | |
| ESM-1 | FALSE | 8 | 1.05 [0.98-1.13] | 1.61E-01 | 3 | 1.07 [0.98-1.17] | 1.20E-01 | 4.36E-01 |
| GAL | FALSE | 1 | 0.90 [0.72-1.11] | 3.28E-01 | 0 | | | |
| hK11 | FALSE | 19 | 1.00 [0.99-1.02] | 7.19E-01 | 5 | 0.99 [0.95-1.03] | 5.72E-01 | 8.54E-02 |
| IL-6RA | FALSE | 92 | 0.99 [0.99-1.00] | 6.61E-02 | 28 | 0.98 [0.97-0.99] | 2.45E-03 | 3.84E-02 |
| IL-8 | FALSE | 4 | 0.87 [0.72-1.04] | 1.14E-01 | 1 | 0.83 [0.68-1.01] | 6.35E-02 | |
| KLK6 | FALSE | 16 | 0.92 [0.88-0.97] | 9.70E-04 | 2 | 0.97 [0.90-1.04] | 3.50E-01 | 9.60E-01 |
| MCP-1 | FALSE | 6 | 0.81 [0.65-1.01] | 5.88E-02 | 2 | 0.91 [0.71-1.18] | 4.83E-01 | 4.70E-01 |
| MMP-1 | FALSE | 83 | 0.98 [0.97-0.99] | 2.68E-03 | 14 | 0.97 [0.94-1.00] | 3.66E-02 | 3.03E-01 |
| MMP-10 | FALSE | 89 | 0.98 [0.97-0.99] | 1.08E-03 | 14 | 0.97 [0.95-1.00] | 4.06E-02 | 5.49E-01 |
| MMP-7 | FALSE | 49 | 1.00 [0.98-1.01] | 8.65E-01 | 7 | 1.01 [0.97-1.04] | 7.08E-01 | 4.50E-01 |
| MPO | FALSE | 30 | 1.10 [1.05-1.14] | 2.53E-05 | 6 | 1.08 [1.00-1.17] | 5.47E-02 | 1.88E-01 |
| PAPPA | FALSE | 7 | 1.05 [0.95-1.16] | 3.06E-01 | 1 | 1.04 [0.86-1.26] | 6.91E-01 | |
| PSGL-1 | FALSE | 27 | 1.00 [0.98-1.02] | 9.15E-01 | 6 | 1.03 [1.00-1.06] | 8.08E-02 | 2.49E-01 |
| PTX3 | FALSE | 11 | 1.02 [0.94-1.10] | 6.04E-01 | 2 | 1.05 [0.93-1.19] | 4.00E-01 | 7.89E-02 |
| SCF | FALSE | 1 | 1.16 [0.85-1.58] | 3.49E-01 | 0 | | | |
| SELE | FALSE | 12 | 1.01 [0.94-1.09] | 7.22E-01 | 1 | 1.01 [0.87-1.18] | 8.57E-01 | |
| SIRT2 | FALSE | 6 | 1.11 [0.99-1.24] | 6.57E-02 | 0 | | | |
| t-PA | FALSE | 1 | 0.91 [0.54-1.54] | 7.26E-01 | 0 | | | |
| TF | FALSE | 49 | 0.95 [0.91-0.98] | 1.10E-03 | 10 | 0.97 [0.92-1.03] | 3.80E-01 | 3.13E-01 |
| TIE2 | FALSE | 69 | 0.98 [0.97-1.00] | 1.75E-02 | 13 | 0.97 [0.94-1.00] | 8.40E-02 | 3.66E-01 |
| TM | FALSE | 50 | 0.98 [0.97-1.00] | 1.74E-02 | 9 | 0.98 [0.95-1.01] | 2.68E-01 | 4.45E-01 |
| TNFSF14 | FALSE | 36 | 1.02 [0.99-1.04] | 1.75E-01 | 7 | 1.02 [0.98-1.06] | 3.92E-01 | 3.23E-01 |
| TRAIL | FALSE | 17 | 1.04 [0.99-1.09] | 1.53E-01 | 3 | 0.98 [0.92-1.05] | 5.92E-01 | 6.85E-01 |
| TRANCE | FALSE | 15 | 0.94 [0.92-0.97] | 1.46E-06 | 3 | 0.98 [0.88-1.09] | 6.75E-01 | 9.38E-01 |

**Supplemental Table 8.** Multiverse sensitivity analysis results for 17 proteins with *cis*-MR associations which survived multiple testing correction

MR = Mendelian randomization; CI = confidence interval; SD = standard deviation; p25 / p50 / p75 = 25th / 50th / 75th percentile

| Protein | Primary MR analysis estimate | MR with strict instrument selection | | | Multiverse MR sensitivity analysis estimates | | | | | | | | | | | Is the estimate robust? |
|---|---|---|---|---|---|---|---|---|---|---|---|---|---|---|---|---|
| | | Estimate | Sign concordance with primary MR analysis | Overlapping 95% CI with primary MR analysis | N | Proportion of positive sign | Proportion of negative sign | Mean | SD | Min | p25 | p50 | p75 | Max | Histogram | |
| ADM | -0.077 | -0.089 | TRUE | TRUE | 111 | 0.00 | 1.00 | -0.086 | 0.019 | -0.133 | -0.096 | -0.088 | -0.071 | -0.036 | 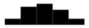 | TRUE |
| CD40 | -0.046 | -0.028 | TRUE | TRUE | 118 | 0.06 | 0.94 | -0.024 | 0.018 | -0.063 | -0.037 | -0.021 | -0.012 | 0.008 | 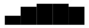 | FALSE |
| CHI3L1 | -0.028 | -0.038 | TRUE | TRUE | 120 | 0.00 | 1.00 | -0.028 | 0.007 | -0.050 | -0.034 | -0.029 | -0.023 | -0.010 | 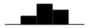 | TRUE |
| CSF-1 | 0.094 | 0.070 | TRUE | TRUE | 109 | 1.00 | 0.00 | 0.090 | 0.025 | 0.050 | 0.073 | 0.084 | 0.103 | 0.157 | 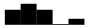 | TRUE |
| CTSL1 | -0.135 | -0.117 | TRUE | TRUE | 84 | 0.00 | 1.00 | -0.123 | 0.025 | -0.189 | -0.134 | -0.119 | -0.110 | -0.066 | 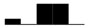 | TRUE |
| CXCL16 | 0.085 | -0.004 | FALSE | TRUE | 112 | 0.93 | 0.07 | 0.047 | 0.037 | -0.062 | 0.017 | 0.045 | 0.068 | 0.143 | 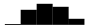 | FALSE |
| EN-RAGE | -0.073 | -0.002 | TRUE | TRUE | 113 | 0.09 | 0.91 | -0.031 | 0.038 | -0.145 | -0.054 | -0.023 | -0.002 | 0.059 | 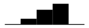 | FALSE |
| FGF-23 | -0.224 | -0.232 | TRUE | TRUE | 83 | 0.00 | 1.00 | -0.182 | 0.033 | -0.265 | -0.201 | -0.180 | -0.161 | -0.097 | 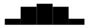 | TRUE |
| Gal-3 | 0.030 | 0.015 | TRUE | TRUE | 112 | 1.00 | 0.00 | 0.027 | 0.011 | 0.011 | 0.017 | 0.026 | 0.032 | 0.057 | 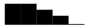 | TRUE |
| HGF | 0.119 | 0.083 | TRUE | TRUE | 113 | 0.97 | 0.03 | 0.071 | 0.035 | -0.019 | 0.045 | 0.075 | 0.094 | 0.147 | 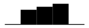 | FALSE |
| HSP | -0.042 | -0.009 | TRUE | TRUE | 110 | 0.19 | 0.81 | -0.012 | 0.015 | -0.050 | -0.021 | -0.013 | -0.004 | 0.029 | 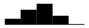 | FALSE |
| KIM-1 | 0.018 | 0.025 | TRUE | TRUE | 111 | 1.00 | 0.00 | 0.017 | 0.009 | 0.002 | 0.010 | 0.016 | 0.021 | 0.040 | 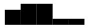 | TRUE |
| MMP-12 | -0.017 | -0.018 | TRUE | TRUE | 102 | 0.00 | 1.00 | -0.020 | 0.009 | -0.045 | -0.024 | -0.018 | -0.013 | 0.000 | 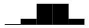 | TRUE |
| MMP-3 | 0.025 | 0.031 | TRUE | TRUE | 112 | 0.94 | 0.06 | 0.024 | 0.012 | -0.002 | 0.017 | 0.024 | 0.032 | 0.052 | 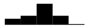 | FALSE |
| OPG | 0.040 | 0.042 | TRUE | TRUE | 112 | 0.94 | 0.06 | 0.043 | 0.028 | -0.024 | 0.027 | 0.040 | 0.060 | 0.105 | 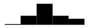 | FALSE |
| PAR-1 | 0.087 | 0.046 | TRUE | TRUE | 112 | 0.98 | 0.02 | 0.072 | 0.033 | -0.010 | 0.052 | 0.065 | 0.093 | 0.156 | 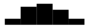 | FALSE |
| SPON1 | -0.051 | -0.055 | TRUE | TRUE | 116 | 0.03 | 0.97 | -0.042 | 0.024 | -0.109 | -0.059 | -0.042 | -0.025 | 0.022 | 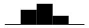 | FALSE |
